# A symbiotic footprint in the plant root microbiome

**DOI:** 10.1186/s40793-023-00521-w

**Published:** 2023-07-31

**Authors:** Kyle Hartman, Marc W. Schmid, Natacha Bodenhausen, S. Franz Bender, Alain Y. Valzano-Held, Klaus Schlaeppi, Marcel G.A. van der Heijden

**Affiliations:** 1 Department of Agroecology and Environment, Plant Soil Interactions, Reckenholzstrasse 191, Agroscope, Zürich, 8046 Switzerland; 2 MWSchmid GmbH, Glarus, 8750 Switzerland; 3 Department of Soil Sciences, Research Institute of Organic Agriculture FiBL, Frick, 5070 Switzerland; 4 Plant Microbe Interactions, Department of Environmental Sciences, University of Basel, Basel, 4056 Switzerland; 5 Institute of Plant Sciences, Faculty of Science, University of Bern, Bern, 3013 Switzerland; 6 Department of Plant and Microbial Biology, University of Zürich, Zürich, 8008 Switzerland

**Keywords:** Arbuscular mycorrhizal fungi, Rhizobia, Symbiosis, Root microbiome, Plant species

## Abstract

**Background:**

A major aim in plant microbiome research is determining the drivers of plant-associated microbial communities. While soil characteristics and host plant identity present key drivers of root microbiome composition, it is still unresolved whether the presence or absence of important plant root symbionts also determines overall microbiome composition. Arbuscular mycorrhizal fungi (AMF) and N-fixing rhizobia bacteria are widespread, beneficial root symbionts that significantly enhance plant nutrition, plant health, and root structure. Thus, we hypothesized that symbiont types define the root microbiome structure.

**Results:**

We grew 17 plant species from five families differing in their symbiotic associations (no symbioses, AMF only, rhizobia only, or AMF and rhizobia) in a greenhouse and used bacterial and fungal amplicon sequencing to characterize their root microbiomes. Although plant phylogeny and species identity were the most important factors determining root microbiome composition, we discovered that the type of symbioses also presented a significant driver of diversity and community composition. We found consistent responses of bacterial phyla, including members of the Acidobacteria, Chlamydiae, Firmicutes, and Verrucomicrobia, to the presence or absence of AMF and rhizobia and identified communities of OTUs specifically enriched in the different symbiotic groups. A total of 80, 75 and 57 bacterial OTUs were specific for plant species without symbiosis, plant species forming associations with AMF or plant species associating with both AMF and rhizobia, respectively. Similarly, 9, 14 and 4 fungal OTUs were specific for these plant symbiont groups. Importantly, these generic symbiosis footprints in microbial community composition were also apparent in absence of the primary symbionts.

**Conclusion:**

Our results reveal that symbiotic associations of the host plant leaves an imprint on the wider root microbiome – which we term the *symbiotype.* These findings suggest the existence of a fundamental assembly principle of root microbiomes, dependent on the symbiotic associations of the host plant.

**Supplementary Information:**

The online version contains supplementary material available at 10.1186/s40793-023-00521-w.

## Background

Plants form diverse associations with microorganisms in, on, and around their roots [1]. The microbes colonizing the root surface or the interior compartment are collectively termed the root microbiome, and there is growing evidence that these microbial associations provide crucial services to plants, enabling their survival in diverse environments and under various biotic and abiotic stress conditions [2, 3]. Despite recent advances in the field, the complex interactions between the different factors determining the composition, diversity, and function of plant-associated microbial communities are still not well understood. Generally, physico-chemical differences in soil, together with the different starting pools of microorganisms that colonize host plant tissues, play a major role in shaping root microbiomes [4–6]. In addition, other factors like plant species, intra-species genetic diversity, and plant evolutionary history further explain differences in composition of root-associated microbiomes [7–9]. However, while such soil and plant effects have received considerable attention, the extent to which root microbiome composition is impacted by the type of symbiotic interactions of a host plant is still poorly understood.

Plants associate with a range of symbionts. Two important groups of plant root-colonizing symbionts, that share a long co-evolutionary history with land plants, are the arbuscular mycorrhizal fungi (AMF) and the N-fixing rhizobia bacteria [10, 11]. AMF form symbiotic associations with 80% of plant families [12] and generally enhance plant performance by providing plants with nutrients especially P and N [13, 14]. In return, the plant provides sugars and lipids to the fungal partners [15]. Rhizobia transform atmospheric N_2_ into plant available ammonia, a limiting nutrient in most terrestrial ecosystems [16, 17]. Although AMF and rhizobia are perhaps the best-known plant-associated symbionts, there is demonstrated variability between different plant families with respect to their associations with these microbes. For example, the nodule-forming symbiosis with rhizobia is more limited and is predominantly found in the plant family of the Fabaceae (legumes) [18], which also establishes symbiosis with AMF. Species within the Poaceae only associate with AMF [19]; whereas some plant families, specifically the Amaranthaceae and Brassicaceae, are considered non-mycorrhizal [19] and also do not associate with rhizobia. Finally, there are exceptions to these generally accepted symbiotic relationships, such as the genus *Lupinus* within the Fabaceae, which associates with rhizobia but not AMF [20].

Association with one, both, or neither of these symbionts could induce direct and indirect changes in the root habitat that could have implications for overall root-associated microbial communities. AMF hyphae, which colonize roots both inter- and extracellularly, have been shown to provide a habitat for a range of associated microorganisms [21–23] and to release hyphal exudates that can affect the hyphal surroundings with potential direct or indirect effects on the abundance and composition of microbial communities [24]. Moreover, AMF hyphae may occupy root spaces that otherwise might be colonized by other endophytic fungi, including some pathogens [25]. Similarly, the symbiosis between host plant and rhizobia results in the formation of root nodules that harbor the N-fixing symbionts [26, 27]. Although they do not induce nodule formation themselves, bacteria of non-N-fixing prokaryotic clades have also been shown to be able to opportunistically colonize existing root nodules and to covary with different locations [28, 29]. Moreover, complex genetic factors and diverse root exudates and secondary metabolites that are important for signaling AMF and rhizobia and inducing colonization of their host plant may also affect other non-target bacteria and fungi in the root microbiome [30–32].

The different strategies plants use to engage in close relationships with primary symbionts (i.e., AMF and rhizobia) raises the fundamental question whether the types of symbiotic associations may contribute to an underlying assembly principle of their wider root microbiomes. Recent works utilizing wild-type (wt) and symbiosis mutants of the legume *Lotus japonicus* have established a clear link between intact genetic regulators of rhizobia and AMF symbiosis and the structuring of the wider root bacterial and fungal microbiome. For example, rhizobia symbiosis mutants of *L. japonicus* differed significantly in the composition of their rhizosphere and endosphere bacteria communities compared to wt plants [33]. Similarly, comparisons of wt and AMF symbiosis mutants showed significant differences in root fungal community composition, as a result of a reduction in AMF taxa and a concomitant increase in Ascomycota in AMF-mutants [34]. However, effects of rhizobia and AMF symbiosis on the wider root microbiome are not limited to within-kingdom interactions. Root bacterial and fungal microbiome comparisons of wt, single, and double AMF/rhizobia mutants have highlighted that AMF symbiosis is necessary for the colonization of specific bacterial taxa in the *L. japonicus* root microbiome, indicative of a multi-kingdom interaction between microbial symbionts and other community members [35].

With one exception [35], recent works examining the effects of AMF and rhizobia symbiosis on the root microbiome have focused on the responses of either bacteria or fungi or only considered a single plant species. Thus, experiments that consider the multi-kingdom effects of AMF and rhizobia symbiosis on the wider root microbiome across a diverse set of plant species are lacking. Additionally, although previous studies have compared root bacterial and fungal microbiomes across a range of plant species and established a clear link between differences in microbiome composition and phylogeny [36–39], these studies did not specifically consider whether the type of symbioses of the tested plant species also influenced the wider root microbiome community structure. Thus, considering root microbiome composition as a function of symbiosis type, plant species identity, and host phylogeny using the resolution of next-generation sequencing could help to reveal the relative importance of these variables, and establish if other root microbiome taxa also change in response to association with AMF and/or rhizobia.

**Fig. 1:**
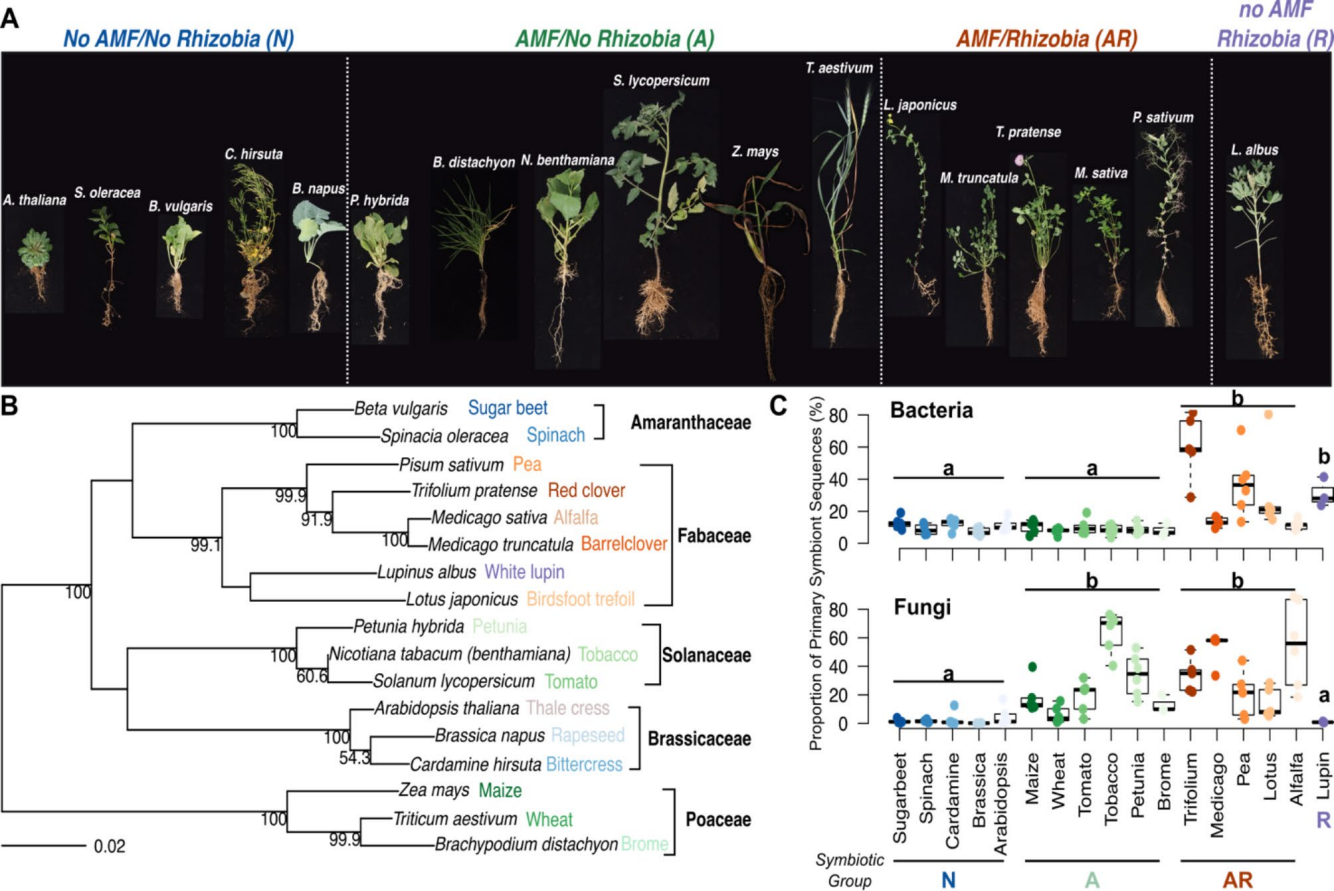
Overview of the 17 plant species used in this study and the proportion of primary symbionts in their microbiomes. **(A)** Photos of individual plants were taken at harvest and are not to scale. Plant species are labeled with their scientific names and are grouped by their known associations with AMF and rhizobia: species without AMF and without rhizobia (*N*), plant species with AMF but without rhizobia (*A*), plant species with AMF and rhizobia (*AR*), and Lupin, which associates with rhizobia but not AMF (*R*). **(B)** The midpoint rooted phylogenetic tree of the 17 species is based on *rbcL* gene sequences obtained from GenBank. Bootstrap values are indicated for nodes with ≥ 50% support in an analysis of 1000 replicates. Because no *rcbL* gene sequence was available for the tobacco species grown in this study (*N. benthamiana*), the sequence of a close relative (*N. tabacum*) was used. Common names for each species are colored by their symbiotype group association in the photograph. Plant families are indicated in black. **(C)** The proportion of sequences of bacterial and fungal primary symbiont origin (see Methods for definition) across the individual species of the different symbiotype groups. Letters indicate significant differences between the symbiotype groups based on the ANOVA results shown in Additional file 1: Table S5

Here, we tested the hypothesis that there is a footprint in the plant root microbiome dependent on the symbiosis type. We specifically tested whether there is a symbiont specific community of root bacteria and root fungi for the different types of symbioses across all plants. For this, we selected 17 different plant species and grouped them according to their symbiosis type with AMF and rhizobia (Fig. 1A & B, Additional file 1: Table S1): plants forming symbiotic associations with AMF and rhizobia (*AR*), plants associating only with AMF (*A*), and plants forming no association with either symbiont (*N*). *Lupinus albus* was included as the only representative of the rare case of plant species forming associations with rhizobia but not AMF (*R*). We cultivated the plants in a standardized soil for ten weeks under controlled greenhouse conditions and profiled the root microbiota using amplicon sequencing. We hypothesized that (i) the root microbiome differs in diversity and structure depending on a species’ symbiosis with AMF and rhizobia, and (ii) we can identify symbiosis-specific community members driven by the type of symbiotic interaction(s) of a host plant.

## Results

### Bacterial and fungal communities in plant roots

Amplification of 16S bacterial rRNA and ITS fungal gene fragments yielded a set of 4,327 and 488 OTUs, respectively (Additional file 1: Fig. S1, Additional files 5 and 6). After removing the primary symbiont OTUs from the dataset (52 OTUs assigned to *Rhizobium*, *Meso*-, *Brady*-, or *Azorhizobium* for 16S; 127 OTUs assigned to *Glomeromycota* (AMF) for ITS, see Methods, Additional file 1: Table S2, Additional file 7), 4,275 16S- and 361 ITS-OTUs remained, respectively. For simplicity, in the following sections we refer to the bacterial and archaeal 16S and fungal ITS OTUs as “b-OTUs” or “f-OTUs”, respectively. Of the 4,275 b-OTUs, 3,617 were classified as bacteria, 1 was classified as Archaea and 657 remained unclassified. Of the 361 f-OTUs, 182 were classified as Fungi and 179 remained unassigned. Taxonomic profiles of the b-OTU and f-OTU communities are presented in Additional file 1: Table S3.

### A symbiotic footprint in microbial communities

First, we confirmed the expected differences in root microbiome composition between the tested plant species of the different symbiosis groups with the primary symbionts included (Additional file 1: Supplementary Results, Figs. S2, S3, Table S4). We then investigated the proportion of sequences belonging to the bacterial and fungal primary symbionts across all plant species. Consistent with the type of symbioses, the different species within group *AR* and Lupin had significantly higher proportions of rhizobia compared to groups *N* and *A* (Fig. 1C, Additional file 1: Table S5). Similar to Garrido-Oter et al., [40], we also found considerable levels of rhizobia reads in non-nodulating plant species with a mean relative abundance (RA) of 10.5% and 9.0% in groups *N* and *A*, respectively. Consistent with microscopy quantification of root AMF colonization of plant species in groups *A* and *AR* (Additional File 1: Fig. S4), the proportion of reads assigned to AMF were significantly higher in groups *A* and *AR*, although with noticeable inter-group variation between plant species (Fig. 1C, Additional file 1: Table S5). Generally, RA of Glomeromycota OTU counts were very low in group *N* and Lupin, comprising a mean of only 2.1% and 0.9% of rarefied counts, respectively.

**Fig. 2:**
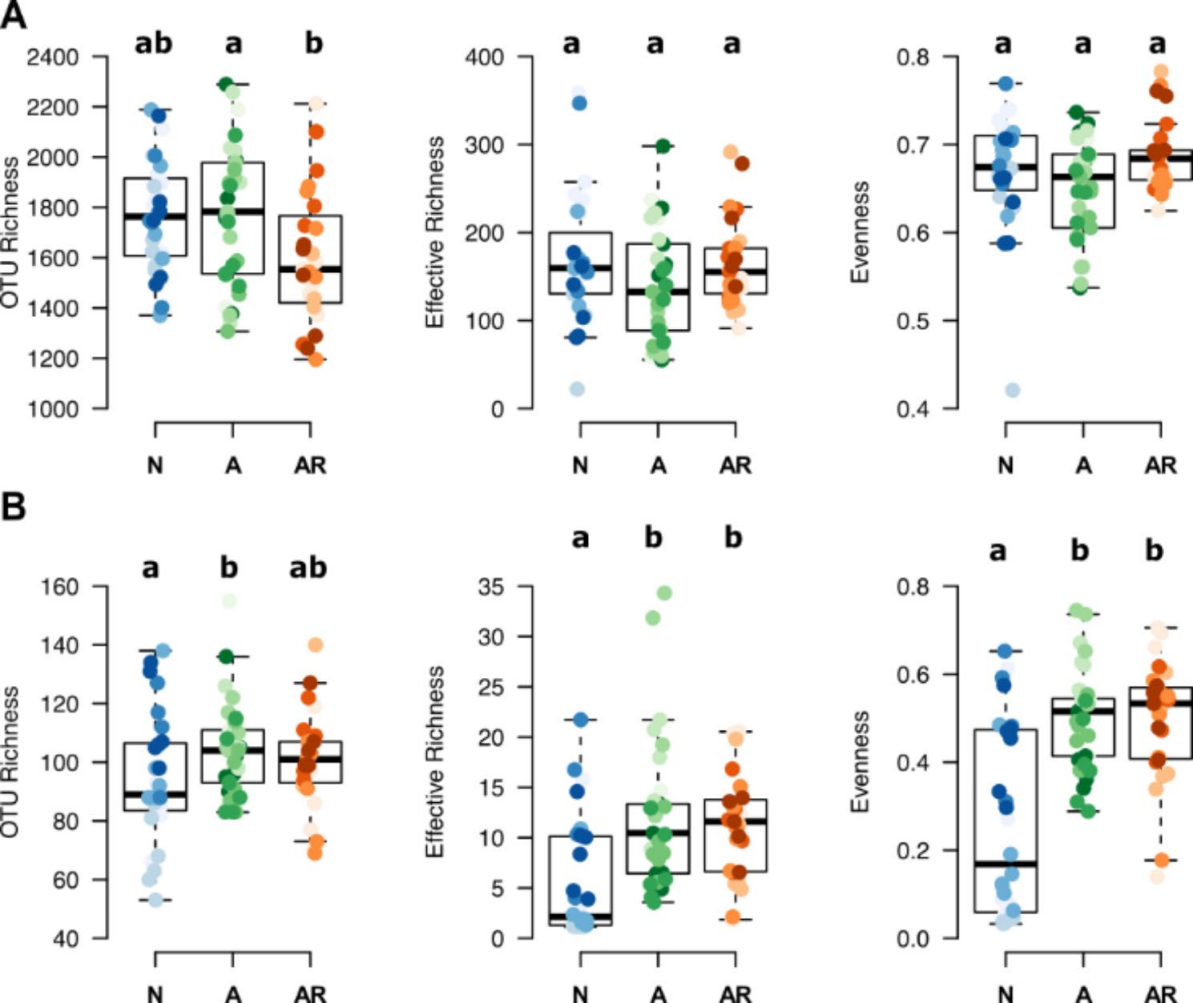
Bacterial and fungal diversity of the symbiosis groups. Boxplots show the observed OTU richness, effective richness, and Pielou’s evenness for the bacteria **(A)** and fungal **(B)** communities with the primary symbiont sequences removed across the symbiosis groups containing multiple species: species without AMF and without rhizobia (*N*), plant species with AMF but without rhizobia (*A*), plant species with AMF and rhizobia (*AR*). Points within the boxes give the individual values per plant species replicate. Letters indicate significant differences between the groups based on the ANOVA results shown in Additional file 1: Table S6. Boxplots for the individual species are shown in Additional file 1: Figure S6. For all boxplots the bottom and top of the boxes correspond to the lower and upper quartiles and the center line marks the median. Whiskers extend to the lowest/highest values unless these values are lower/higher than the first/third quartile minus/plus 1.5 times the inner quartile range, which equals the third minus the first quartile

To specifically investigate the effects of symbiosis type on the wider microbiome, we removed primary symbiont reads from the data for all further analyses (see Methods) and rarefied the data to the sample with the lowest number of counts in each dataset (10,882 and 1,090 sequences per sample for 16S and ITS, respectively). This was sufficient to capture most of the profiled diversity (Additional file 1: Fig. S5). We then assessed whether symbiosis type, plant family, and plant species identity affected microbiome diversity by analyzing b-OTU and f-OTU observed richness, effective richness (exponent of the Shannon index), and evenness (Additional file 1: Fig. S1) between the three symbiosis groups (Fig. 2) plant families within groups *A* and *N*, and plant species within groups *A*, *AR*, and *N* (Additional file 1: Fig. S6). In the bacterial community, only b-OTU richness was significantly affected by the symbiosis group, with significantly higher richness in group *A* compared to *AR* (Fig. 2A, Additional file 1: Table S6). In the fungal community, we observed significantly lower f-OTU richness, effective richness, and evenness in group *N* compared to the other groups, which were not significantly different from each other (Fig. 2B, Additional file 1: Table S6).

Plant family and species effects on b-OTU diversity measures were limited to significant differences in all diversity measures between plant species within group *N* and differences in evenness between plant species within group *A* (Additional file 1: Fig S6, Table S6). In the f-OTU community, we noted significant differences between plant families within group *N* (Amaranthaceae vs. Brassicaceae) for all diversity measures and differences between group *A* families (Poaceae vs. Solanaceae) for effective richness (Additional file 1: Table S6). Effects of the individual plant species were limited to differences in observed richness between species in group *N* and effective richness between species in group *A* (Additional file 1: Fig S6, Table S6).

**Fig. 3:**
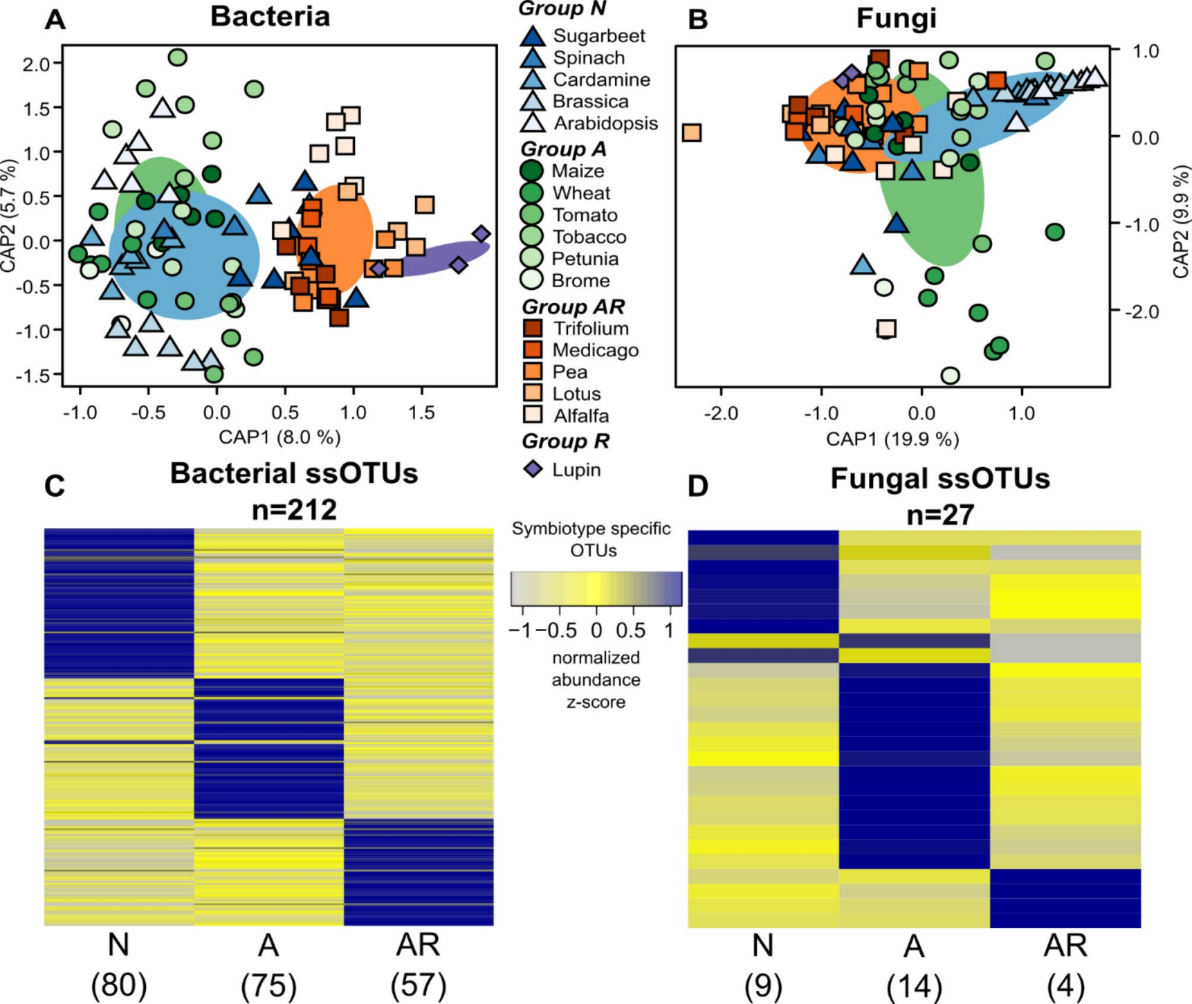
Symbiotype groups harbor unique communities of bacteria and fungi. Constrained analysis of principal coordinates (CAP) on Bray-Curtis dissimilarities of all samples analyzed and the factors presence/absence of rhizobia, presence/absence of AMF, and plant species as explanatory terms in bacterial **(A)** and fungal **(B)** datasets without the primary symbionts. The percentage of total variance explained by each CAP axis is given in parentheses. Ellipses represent the 95% confidence interval of the group centroids. Heatmap values reflect the z-scores of the DESeq2 normalized OTU abundance values for the bacterial **(C)** and fungal **(D)** symbiotype specific OTU communities for symbiotypes *N*, *A*, and *AR*. The total number of enriched OTUs (log_2_FC > 1, adjusted *p* (FDR) < 0.05) for each symbiotype is indicated below each symbiotype name. Taxonomy assignments and normalized abundance values of all ssOTUs in the individual plant species and symbiotype groups are presented in Additional file 8

Next, we evaluated the overall differences in community composition between microbiomes of the different symbiotic groups. For this, we conducted CAP ordinations on Bray-Curtis dissimilarities calculated using the rarefied b-OTU and f-OTU abundances with the primary symbionts removed and the factors presence/absence of rhizobia and presence/absence of AMF as explanatory terms (Fig. 3A & B). Presence/absence of rhizobia appeared to drive differences in the b-OTU community, with Groups *N* and *A* clustering together, and separately from, Group *AR* and Lupin. The primary CAP axes explained 13.7% of overall variance (Fig. 3A). In the f-OTU community, the clustering of samples by the presence or absence of the symbionts was less apparent. However, the primary axes explained 29.8% of the overall variance in the community (Fig. 3B). Unconstrained PCoA ordinations revealed similar patterns as the CAP analyses, highlighting the differences in root microbiome composition between legumes and non-legumes in the b-OTU community and species from group *N* compared to the other species in the f-OTU community (Additional file 1: Figure S7).

Taken together, this would suggest that root microbiomes exhibit a characteristic footprint for the type of symbioses a host plant engages with. Importantly, this is not only based on primary symbionts but is determined by the other community members. We refer to these footprints as ‘symbiotypes’ of root microbiomes that reflect the type of symbioses (*N*, *A*, and *AR* interactions) of the host plant.

### Relative effect sizes of symbiotype and plant species driving microbiome composition

Next, we quantified the effect sizes of the different symbiotic groups on b-OTUs and f-OTUs community composition using PERMANOVA (Table 1). This analysis corroborated the symbiotypes, with a significant symbiotic group effect explaining 7.4% of variance (in terms of % SS) in the b-OTUs and 13.8% in the f-OTUs (Table 1).The effects of differences between plant families and species generally demonstrated the largest effect size (in terms of combined % SS) in determining differences in community compositions across both microbial kingdoms (Table 1). We noted significant differences between families in groups *A* and *N*, which explained a combined proportion of variance similar to the symbiotype effect – 6.9% vs. 7.4%, respectively (Table 1). Differences between plant species from groups *A, AR*, and *N* was the strongest determinant of b-OTU community composition, explaining a total of 20.8% of overall variance (Table 1). In the f-OTU community, significant differences between families in groups *A* and *N* and species in groups *A* and *AR* explained a combined 15.8% and 13.9% of variance respectively, which was more than the symbiotype effect (13.8%; Table 1). Furthermore, Mantel tests revealed significant, positive correlations between plant species phylogenetic relatedness and b-OTU (Mantel’s ρ = 0.31, p = 0.0001) and f-OTU (Mantel’s ρ = 0.38, p = 0.0001) community dissimilarity.

In short, we confirmed that plant family and species identity is a significant driver of differences in root microbiome composition, which was also positively correlated with the phylogenetic relatedness between plant species. However, the effect of symbiosis type also presents a driver of root microbiome composition, and a similarly strong effect of symbiosis type when the primary symbionts were removed furthers supports the concept of a symbiotype.

**Table 1:** PERMANOVA testing for the effects of symbiotic groups, plant family, and plant species on community composition. Tests were conducted on Bray-Curtis dissimilarities of bacterial and fungal OTU communities with the primary symbiont OTUs removed. For the term “Symbiotic Group” three different contrasts testing all possible combinations of factor levels of symbiotic groups against each other were performed (a, b, c)

Bacteria	df	F	*p*	%-SS	Fungi	F	*p*	%-SS
seqDepth	1	2.16	***0.001***	1.71	seqDepth	11.71	***0.001***	6.81
Pot	1	4.61	***0.001***	3.65	Pot	7.70	***0.001***	4.48
isLupin	1	3.23	***0.001***	2.56	isLupin	3.21	***0.007***	1.87
Symbiotic Group	2	4.66	***0.001***	7.38	Symbiotic Group	11.84	***0.001***	13.77
*a) A vs. others*	1	4.07	***0.001***	3.22	*a) A vs. others*	5.73	***0.001***	3.33
*a) AR vs. N*	1	5.26	***0.001***	4.16	*a) AR vs. N*	17.94	***0.001***	10.43
*b) AR vs. others*	1	6.68	***0.001***	5.28	*b) AR vs. others*	14.35	***0.001***	8.34
*b) A vs. N*	1	2.65	***0.001***	2.1	*b) A vs. N*	9.33	***0.001***	5.42
*c) N vs. others*	1	3.91	***0.001***	3.09	*c) N vs. others*	16.54	***0.001***	9.62
*c) AR vs. A*	1	5.42	***0.001***	4.29	*c) AR vs. A*	7.13	***0.001***	4.15
Group *A* families (Poaceae vs. Solanaceae)	1	5.56	***0.001***	4.4	Group *A* families (Poaceae vs. Solanaceae)	9.76	***0.001***	5.67
Group *N* families (Amaranthaceae vs. Brassicaceae)	1	3.21	***0.001***	2.54	Group *N* families (Amaranthaceae vs. Brassicaceae)	17.45	***0.001***	10.15
Group *A* plant sp.	4	2.91	***0.001***	9.22	Group *A* plant sp.	4.09	***0.001***	9.51
Group *AR* plant sp.	4	1.91	***0.001***	6.03	Group *AR* plant sp.	1.89	***0.006***	4.4
Group *N* plant sp.	3	2.33	***0.001***	5.53	Group *N* plant sp.	0.84	0.654	1.47
Residuals	72			56.98	Residuals			41.87

**Fig. 4:**
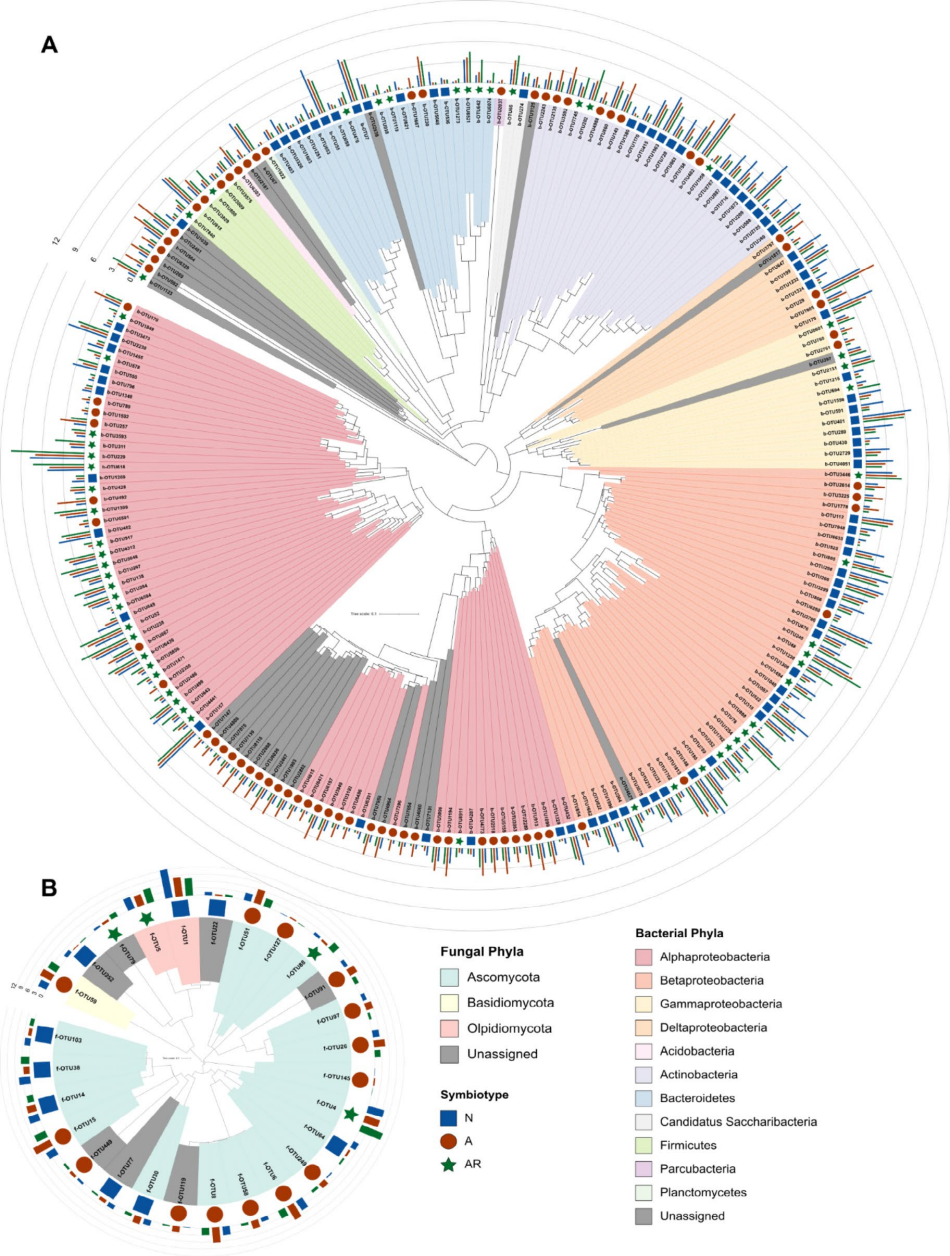
Symbiotype membership shown as taxonomic diversity of the bacterial and fungal symbiotype specific ss-OTUs. Phylogenetic tree of the symbiosis specific ss-OTUs in the **(A)** bacteria community (n = 212) and **(B)** fungal community (n = 27). Tree branches are colored by OTU phylum assignment, with Proteobacteria in the bacteria community being further separated into class. Colored shapes (square, circle or star) indicate the symbiotype membership for each OTU. The bar graphs show the mean abundance values of each OTU within each symbiotype. Abundances of the ss-OTUs across the individual plant species and full taxonomy assignments are presented in Additional file 8

### Symbiotype-specific OTUs

To delineate the bacteria and fungi that were specific for the symbiotypes of the root microbiomes, we used DESeq2 (see Methods) to test for differential abundance between plant species grouped by their symbiosis types. We then tested for enrichment or depletion of the different taxa, and trophic mode annotations from FUNGuild in the f-OTU community, in the differentially abundant OTUs.

Of the 4,275 b-OTUs and 361 f-OTUs in the analysis, 953 (22.3%) and 83 (23%) unique OTUs were significantly differentially abundant (adjusted *p* (FDR) < 0.05, log_2_ FC ≥ 1) between the different symbiotypes, respectively (Additional file 1: Table S7). This high proportion of symbiotype-responsive microbes highlights the broad impact that the presence or absence of AMF and rhizobia have on the abundance of individual OTUs from both kingdoms.

Most notably, in the b-OTU community, the Firmicutes were depleted in the community of OTUs that were negatively affected by a host species’ association with AMF only (Table 2). Additionally, we noted the community of differentially abundant b-OTUs between species forming symbioses with both AMF and rhizobia or neither symbiont was depleted in the bacterial phyla Acidobacteria, Chlamydiae, and Verrucomicrobia (Table 2). Interestingly, however, we did not detect any enriched or depleted fungal taxa in the differentially abundant fungal OTUs (Table 2).

**Table 2:** Enriched and depleted taxa in the differentially abundant OTUs. Enrichment or depletion of taxa at different taxonomic levels within the OTUs that were identified as differentially abundant between plant symbiosis types (see Additional file 1: Table S7) or specifically enriched in symbiotype groups *N*, *A*, or *AR* (see Fig. 3 C & D, Fig. 4, Additional file 1: Table S8). The column “Type” indicates the direction of the relationship and the set of OTUs in which it occurs: The suffix “General” refers to all OTUs that are significant in the indicated contrast, i.e., set union of all OTUs significantly more or less abundant. “Up” refers to OTUs that are significantly more abundant in the first group of the contrast; whereas “Down” refers to OTUs that are significantly less abundant in the first group of the contrast. “Enrichment” indicates that the indicated taxon is significantly more frequent in the set of OTUs tested. “Depletion” indicates that the taxon is significantly less frequent in the set of OTUs tested. Observed and expected values are based on a 2 × 2 contingency table of OTU counts with Fisher’s Exact Test *p*-values FDR corrected for multiple testing (see Methods, Additional file 1: Fig. S1)

Bacterial OTUs						
Contrast or Group	Type	Taxonomic Level	Taxon	Observed	Expected	P-value (FDR)
**Differentially abundant by plant species symbiotic group**						
A vs. N	Depletion Down	Phylum	Firmicutes	0	7.1326	0.0078
AR vs. N	Depletion General		Acidobacteria	4	18.8632	0.0009
AR vs. N	Depletion General		Verrucomicrobia	3	11.3179	0.0470
AR vs. N	Depletion General		Chlamydiae	2	9.0779	0.0496
AR vs. N	Depletion Down		Acidobacteria	1	12.9871	0.0006
AR vs. N	Enrichment Down		Proteobacteria	187	151.7060	0.0016
AR vs. N	Depletion Up		Actinobacteria	8	24.0550	0.0014
AR vs. A	Depletion General		Chlamydiae	0	7.6009	0.0103
AR vs. A	Depletion General		Firmicutes	11	24.8758	0.0182
AR vs. A	Depletion General		Acidobacteria	5	15.7942	0.0182
AR vs. A	Depletion General		Verrucomicrobia	2	9.4765	0.0442
AR vs. A	Enrichment Down		Actinobacteria	75	47.3439	0.0015
AR vs. A	Depletion Up		Actinobacteria	3	17.3135	0.0002
**Symbiotic Group Enriched OTUs**						
Group N	Enrichment	Phylum	Proteobacteria	52	34.9754	0.0023
Group N	Enrichment	Class	Betaproteobacteria	24	8.1965	0.0001
Group N	Enrichment		Flavobacteriia	8	0.9357	0.0002
Group N	Enrichment	Order	Burkholderiales	21	5.8760	0.0000
Group N	Enrichment		Flavobacteriales	8	0.9357	0.0002
Group N	Enrichment	Family	*Flavobacteriaceae*	8	0.8421	0.0003
Group N	Enrichment		*Micromonosporaceae*	7	0.8234	0.0016
Group N	Enrichment		*Oxalobacteraceae*	10	2.2643	0.0044
Group N	Enrichment		*Xanthomonadaceae*	7	1.4222	0.0220
Group AR	Enrichment	Phylum	Proteobacteria	43	24.9200	0.0000
Group AR	Enrichment	Class	*Alphaproteobacteria*	25	11.9200	0.0048
Group AR	Enrichment	Genus	*Novosphingobium*	5	0.3200	0.0086
**Fungal OTUs**						
*None detected*						

We also identified b-OTUs and f-OTUs that were specific for each of the three symbiotypes – *N*, *A*, and *AR* (log_2_ FC > 1, adjusted *p* (FDR) < 0.05; Fig. 3C & D). In total, we found 80, 75, and 57 bacterial symbiotype-specific OTUs (ss-OTUs) in groups *N*, *A*, and *AR*, respectively (Figs. 3C and 4A, Additional file 1: Table S8, Additional file 8). These identified ss-OTUs comprised a total of 10.8%, 10.6%, and 12.1% of rarefied sequences in the symbiotypes *N*, *A*, and *AR*, respectively. We found that 21% of the ss-OTUs in group *AR* belonged to the order Rhizobiales (Additional file 1: Table S8). However, we noted ss-OTUs in group *AR* were only significantly enriched for Alphaproteobacteria, particularly the genus *Novosphingobium* (Table 2). The ss-OTUs for group *A* also comprised the Rhizobiales (12%) with a larger proportion of unclassified OTUs (~ 21%). From group *N*, we noted that many of the ss-OTUs (~ 26%) belonged to the order Burkholderiales from the Proteobacteria, which was significantly enriched, particularly for the family *Oxalobacteraceae* (Table 2, Additional file 1: Table S8). Approximately 18% of ss-OTUs in group *N* belonged to the order Actinomycetales from the phylum Actinobacteria (Fig. 4; Table 2; Additional file 1: Table S8); however, we also noted other significantly enriched taxonomic groups, including the family *Flavobacteriaceae* from the order Flavobacteriales, as well as the families *Micromonosporaceae* and *Xanthomonadaceae* (Table 2).

We found fewer ss-OTUs in the fungal community, with groups *N*, *A*, and *AR* having 9, 14, and 4 ss-OTUs, respectively (Figs. 3D and 4B, Additional file 1: Table S8, Additional file 8). These ss-OTUs comprised 65.9%, 27.3%, and 25.5% of rarefied sequences in their respective symbiotypes. However, we did not detect any consistent enrichment patterns of taxonomic groups in the fungal ss-OTUs (Table 2), and 50% of the OTUs enriched in groups *A* and *AR* could not be taxonomically assigned at any level, likely the result of employing a high taxonomy assignment confidence threshold (Additional file 1: Supplementary Methods, Table S8).

Overall, bacterial taxa were more responsive to the presence or absence of rhizobia and AMF than fungal taxa, as we did not detect any specific taxonomic responses to the presence or absence of AMF and rhizobia in the fungal community. Across bacterial taxa, only the Firmicutes were significantly less likely to be affected by the presence or absence of AMF, but multiple phyla were significantly more likely to be affected by the presence or absence of AMF and rhizobia, including Acidobacteria, Actinobacteria, Chlamydiae, and Verrucomicrobia.

## Discussion

Rhizobia and AMF are two of the most common plant-symbiotic organisms associating with over 80% of all plant species. It is still unresolved whether relationships with these important microbes entail a symbiont footprint in the overall root microbiome composition. Here, we have used bacterial and fungal profiling of root samples from 17 different plant species forming different combinations of symbiotic associations with AMF and rhizobia to address how these associations affect the diversity and community composition of the wider (i.e., without primary symbionts) root and rhizosphere microbiomes. We hypothesized that the rhizosphere microbiome differs in community structure and diversity depending on the type of symbioses of a host plant and that we could identify a symbiosis-specific footprint in plant root microbiomes.

We first confirmed that differences between host plant family and species identity and phylogeny were the most influential drivers of bacterial and fungal diversity and community composition (Table 1, Additional file 1: Table S4). This is consistent with previous studies showing host plant species effects, at least on root bacteria community composition, become more influential in closer proximity to the root [7, 8, 36] and become stronger when more distantly related species are compared [8, 37, 41]. Moreover, we found that host plant species and phylogeny were highly influential in determining fungal community composition, in accordance with previous work investigating plant species effects on fungal communities profiled from the same or physicochemically similar soils [42, 43]. Thus, our results highlight that like bacterial communities [7, 44], root fungal community composition is also correlated with host phylogeny.

It is well known that other plant traits (e.g., specific root length or root diameter) that likely co-vary with symbiosis type also influence root microbiome composition [reviewed in 45]. For instance, a recent study demonstrated that grasses have a higher specific root length (e.g., longer roots per gram root) compared to herbs and legumes [46]. However, as most of the plant species included in our present study are herbs and legumes, it is less likely that differences in specific root lengths were a major driver of changes in microbiome composition. Furthermore, both the non-mycorrhizal and the mycorrhizal traits are present in phylogenetically diverse plant groups (see Fig. 1), and it is likely that non-mycorrhizal and mycorrhizal plants are highly divergent in other traits as well. Hence, the detection of generic symbiosis footprints in microbial community composition - despite the many differences in plant traits (e.g., root morphology), phylogeny, and life history (i.e., annuals vs. perennials) among the tested plant species supports our hypothesis that the type of symbioses is an important driver of the plant root microbiome. This is indicated by the symbiont-specific footprint – referred to as ‘symbiotype’ – in root microbiome composition (Figs. 3 and 4). The symbiotype includes additional microbial community members that differ together with the primary symbionts (i.e., AMF or rhizobia) in the root-associated microbiomes of the symbiotic groups as revealed by ordination (Fig. 3A & B) and PERMANOVA (Table 1) analyses. In addition, we detected a range of symbiosis-type-specific microbial taxa (Fig. 4). Together these results show that associations with AMF and rhizobia also affect the composition of the wider microbiome and that root microbiomes have a discernible symbiotype footprint reflecting the type of symbiotic interactions a host plant engages with. Although here we have considered the type of symbioses qualitatively, it will be interesting to further investigate how possible differences in the symbiotic proficiency of the primary symbionts in these plant species is reflected in the wider root microbiome.

In the case of association with rhizobia, the major changes to root morphology include the formation of nodules inhabited by rhizobia engaged in N-fixing activities. As such, plants associating with rhizobia exhibit higher plant N concentrations compared to other plants [47], which could indirectly shift root microbiome composition as a result of reduced nutrient stress or enhanced plant immunity. However, other, presumably non-N-fixing, prokaryotic clades have also been shown to be present in root nodules [33, 48]. Moreover, experiments with nodulation pathway mutants of *L. japonicus* suggested that rhizobia may act as modulating microbes, and the presence and proliferation of other, non-N fixing microbes in legumes could be a result of microbe-microbe interactions or the ability of non-rhizobia to exploit plant nodulation signals [33]. Similarly, AMF hyphae have been shown to provide a habitat for a range of specialized bacteria which colonize both the interior and surface of AMF hyphae [21, 22, 49]. AMF also release hyphal exudates, which alter the surrounding pH, nutrient, and C availability, inducing shifts in abundance and composition of hyphae-associated microbial communities [50, 51]. Additionally, recent work with AMF symbiont mutants of *L. japonicus* highlighted that, similarly to the importance of rhizobia symbiosis genes in structuring the non-rhizobia bacteria community [33], the presence of certain bacterial taxa in root samples is dependent on root colonization of AMF, indicative of a multi-kingdom interaction mediated by AMF symbiosis [35].

Plants substantially alter the diversity and composition of their root microbiomes through secretion of root exudates [52]. Differences in root exudation patterns among the species of the different symbiotic groups likely played a role in recruiting the primary symbionts but also shaping the wider root microbiome. Exudate compounds such as plant hormones like strigolactones initiate hyphal branching, AMF recruitment, and the subsequent inter- and extracellular root colonization by the symbionts [53]. Recent evidence also suggests that strigolactones have both positive and negative impacts on the growth of other, non-mycorrhizal fungi associating with plant roots [30, 32]. Similarly, the symbiosis between host plant and rhizobia has been shown to be initiated by plant exudation of flavonoids, a diverse set of plant secondary metabolites important for symbiont recruitment and the formation of root nodules [26, 27]. It has also been suggested that flavonoids are important for AMF symbiosis signaling [31] and may contribute to driving community dynamics of other bacteria and fungi in the root microbiome [54]. It is also possible that plant metabolites, which are not linked to the recruitment of the primary symbionts, could have played a role in our observation of significantly lower fungal diversity measures in group *N* (Fig. 2B). For example, species within the Brassicaceae are well characterized for their production of glucosinolates, a class of sulfur-rich secondary metabolites [55, 56]. Glucosinolates – enzymatically activated to isothiocyanates [55] – have been shown to alter the community composition of root-associated fungal communities in the Brassicaceae species *A. thaliana* [57], and to reduce fungal richness and diversity when applied exogenously to soil [58, 59]. Interestingly, we noted the lowest intra-group fungal diversity measures in group *N* for the Brassicaceae species (Additional file 1: Fig. S6B), supporting the idea that fungi may be more susceptible to isothiocyanates than bacteria [60].

In short, differences in the wider microbiome of the tested plant species that varied in their associations with AMF and rhizobia are likely the result of a combination of differences in the known microbial communities that associate with the different symbionts, notably AMF hyphae, indirect responses to changes in root morphology and physiology, or as a result of exudation patterns in response to symbiotic type. Further work is necessary to experimentally test which factors change the root microbiome and how this affects plant functionality. Moreover, while our results are indicative for the existence of the symbiotype, future studies that compare AMF and/or rhizobia mutant and non-mutant plants are required to experimentally confirm the symbiotype effect on root microbiome composition. Although symbiotic mutants exist for several of the investigated plant species, a rigorous disentangling of the symbiotype effect from the influences of host plant species and phylogeny requires symbiotic mutants in all plant species. Such experiments would permit a generalization of the symbiotype effect.

Further support for our hypothesis that there is a symbiotic specific community of bacteria and fungi comes from our finding that a specific set of b-OTUs and f-OTUs were significantly enriched in *N*, *A*, and *AR* groups (Fig. 4, Additional file 1: Table S8). Additionally, we noted several intriguing taxonomic trends. For instance, Firmicutes were more abundant in the presence of AMFs (Table 2). Certain taxa within the Firmicutes have been reported to respond positively to the presence of AMF [61, 62]. However, our results indicate little interaction between Firmicutes and AMF, suggesting the nature of the relationship could be soil-type and/or growth condition specific.

We also found a significant depletion of Acidobacteria, Chlamydiae, and Verrucomicrobia in the community of differentially abundant OTUs between groups *AR* and *N* (Table 2), indicative of limited interactions between these phyla and the primary symbionts. Acidobacteria are typically abundant in bulk soil compared to root-associated communities [62–64] and primarily respond to changes in pH [65] and soil nutrient availability [66, 67]. Members of the Chlamydiae have been reported in root-associated communities of maize [68], poplar [69], and tobacco [70]. However, little is known about the phylum’s functional relationship with plants [71], and thus their role in the plant root microbiome and the factors determining community assembly and dynamics certainly warrants further exploration. The Verrucomicrobia were once thought to be relatively rare in environmental samples due to primer biases that exclude the phylum [72]. However, their detection in the root microbiomes of various plant species [68, 73, 74] supports the idea that the abundance of Verrucomicrobia in the root microbiome may be greater than originally thought [75]. While it is also possible that our primers also underestimate the frequency of Verrucomicrobia in our samples too, our results hint at a more generalist lifestyle of some Verrucomicrobia taxa and that they are less responsive to different plant symbiosis types.

Interestingly, various OTUs from the Rhizobiales were enriched in groups *N* and *A*, comprising plant species not associating with N-fixing rhizobia. Members of the Rhizobiales have been reported in a variety of environments [76] and possess other life history strategies including parasitism and free-living lifestyles [76, 77]. However, given their consistent enrichment in the root microbiomes of both legume and non-legumes, Rhizobiales are likely members of a core root microbiome common to a wide variety of plant species [40]. Similarly, members of the other enriched taxa detected primarily in group *N* are hypothesized to be involved in several important plant functions. For example, we detected ss-OTUs from the Actinobacteria, which are commonly reported in root microbiome surveys, and a wide body of recent studies revealed they are enriched under drought conditions [78]. Additionally, members of the Burkholderiales, a class enriched in group *N*, have been proposed to be keystone taxa, suggesting they may play important roles in mediating the structure and function of the wider root microbiome [79].

Finally, while it is tempting to speculate on the putative role of all the enriched bacterial and fungal taxa we detected, we recognize that the ultimate functions of individual root microbiome members and the wider community are controlled by a complex interplay of host plant and microbe molecular, genetic, and biochemical factors that are not captured by the marker gene sequencing we have conducted. As such, more targeted investigations, like comparative analyses of root exudate chemistry or host-microbe transcriptomic profiling, are needed to elucidate the nature of the relationships between these root community members enriched in the different symbiotic groups and their plant hosts.

## Conclusion

Despite associating with a wide variety of plant species, our understanding of how symbiosis with rhizobia and AMF influences the wider root microbiome remained limited. Here we revealed the existence of symbiotypes – characteristic compositions of the root microbiome – which reflect the type of symbioses of the host plant. The symbiotypes are apparent despite the effects of plant family and species identity and phylogeny. This symbiosis-dependent signature in root microbiome communities is reminiscent of the concept of enterotypes. Enterotypes were defined as microbiome signatures that stratify clusters of humans based on similar compositions of their gut microbiomes [80]. Although the discrete enterotypes and their drivers remain contested [81, 82], long-term diet has been associated as one driver of enterotypes [83]. A plant’s ‘diet’ is the surrounding soil, and plants rely on different nutrient acquisition strategies for their ‘dietary intake’. Symbiotic plants such as legumes and mycorrhizal hosts rely largely, but not exclusively, on microbially provided N and P, respectively, whereas non-symbiotic plants rely on one’s own nutrient acquisition traits. Hence, like the idea of diet-driven enterotypes, the different ‘nutritional modes’ of plant groups (*N, A, AR*) may explain the identified symbiotypes that stratify clusters of root microbiomes based upon the type of symbiotic interactions of the hosts. Expanding on this idea would suggest that the symbiotypes are derived observations reflecting the ‘nutritional modes’ of symbiosis groups (*N, A, AR*); whereas the host plant nutrient homeostasis might be the proximal driver of root microbiome assembly.

## Methods

A detailed description of the methods used in this study can be found in Additional file 1: Supplementary Methods.

### Plant growth and sample collection

Seeds of 17 different plant species from five families were sown into replicate pots (3–6 replicates per species, Additional file 1: Table S1) containing a non-sterile sand/soil mixture and grown in a greenhouse for 10 weeks before sampling of the root-associated microbiota following Bodenhausen *et al.,* [84]. For legume species (Additional file 1: Table S1), we prepared additional nodule samples to define the primary symbiont rhizobia species. Because our root microbiota sampling method did not discriminate between rhizoplane (root surface) or endophytic (root interior) compartments, we refer generally to the sampled unit as “root-associated” or “root” microbiota.

### PCR and sequencing library preparation

DNA was extracted from roots and separate nodule samples and 16S rRNA and ITS gene amplicon sequencing libraries generated using the PCR primers 799F [85] and 1193R [86], and ITS1F [87] and ITS2 [88], respectively. The primers were extended at the 5’end with an error-tolerant barcode for multiplexed library sequencing (Additional file 2). The MiSeq libraries were prepared and sequenced at the Functional Genomics Center Zürich (www.fgcz.ch).

### Bioinformatics and sequence processing

Operational taxonomic units (OTUs) were generated with UPARSE [89] (usearch v10.0.024). For the ITS data, the ITS1 sub region was extracted with ITSx [90] (v1.1.1). Sequences were clustered with a minimal identity threshold of 99% to obtain 6,052 16S- and 706 ITS-OTU sequences (Additional files 3 and 4, Additional file 1: Fig. S1). The 16S-OTU sequences were annotated with the Ribosomal Database Project taxonomy database [91] (v16). The ITS-OTU sequences were annotated with a combination of the PLANiTS database [92] (Status March 2020) and the UNITE database [93] (v8.3) with the usearch command *sintax* [94] (v10.0.240). Any OTU sequences classified as Viridiplantae were removed. However, we retained OTUs whose taxonomy was “Unassigned” for all analyses, as this was likely the result of employing a high confidence threshold (0.8) for taxonomy assignment. ITS-OTUs were further annotated with functional categories using FUNGuild [95] (v1.1). Finally, to avoid sequencing artifacts, OTU sequences with less than 30 (16S) or 10 (ITS) counts in total or with counts in less than three samples were removed from all further analyses. As a result, 4,327 bacterial and 488 fungal OTUs remained after this filter (Additional files 5 and 6, Additional file 1: Fig. S1).

### Identification of primary symbionts

We used two approaches to identify the primary symbionts (i.e., rhizobia and AMF) in the root microbiome datasets. For the 16S-OTUs we defined primary symbionts based on a combination of the taxonomic identity of well-known nodule-colonizing bacteria and a sequence count threshold of OTUs from these taxa in nodule samples. More specifically, we marked 16S-OTUs annotated as *Rhizobium*, *Mesorhizobium*, *Bradyrhizobium* or *Azorhizobium* with at least 10 raw reads across the individually sequenced nodule samples collected from the legume species at harvest as nodule-specific, symbiotic OTUs (52 OTUs). In the ITS-OTU community, OTUs annotated as Glomeromycota in root samples of all plant species were marked as specific AMF OTUs (127 OTUs). We refer to these nodule- and AMF-specific OTUs collectively as ‘primary symbionts’, and for specific analyses – i.e., to test whether changes in the root microbiome are also detected without these taxa – these OTUs were removed from their respective datasets (Additional file 7, Additional file 1: Fig. S1; Table S2).

### Analysis of diversity, between sample distances, and community structure

All statistical analyses were performed in R [96]. Counts of the filtered OTU sequences were rarefied to the sample with the lowest number of counts in the dataset. This was sufficient to capture most of the profiled diversity (Additional file 1: Fig. S5). For calculating and testing effects of the symbiotic groups on diversity indices, we analyzed the variation in OTU observed richness, effective richness (exponent of the Shannon index [97], and evenness [98] calculated on rarefied OTU counts with linear ANOVA models. Bray-Curtis dissimilarities between all samples containing the filtered OTUs were visualized using constrained analysis of principal coordinates (CAP) with the R package *vegan* [99]. We assessed the impact of the symbiotic groups on community structure with a permutational multivariate ANOVA (PERMANOVA) on Bray-Curtis dissimilarities calculated from rarefied datasets with and without the primary symbionts. The structure of all (PERM)ANOVA models followed general design principles (see Schmid *et al.*, [100] for a detailed discussion of this approach), and factors were fitted sequentially (type I sum of squares). Significance tests were based on *F*-tests calculated manually using appropriate error terms and denominator degrees of freedom.

### Phylogenetic analyses between plant species and community composition

Phylogenetic relationships between the 17 plant species were determined based on chloroplast *rbcL* gene sequences [101] obtained from GenBank (Additional file 1: Table S1), aligned using the R package *msa* [102], and visualized as a phylogenetic tree with the R package *phangorn* [103]. To assess if pairwise distances in bacterial and fungal community composition between samples were correlated with phylogenetic similarity among plants, we performed a Mantel test on a phylogenetic distance matrix of the investigated plant species and a Bray-Curtis dissimilarity matrix of the bacterial or fungal communities. The pairwise evolutionary distance matrix of the plant species was calculated with the R package *ape* [104].

### Identification of differentially abundant OTUs

Variation in individual OTU relative abundances were analyzed with a generalized linear model using the R package *DESeq2* [105] according to factorial designs. Although originally developed for gene expression analyses, tools like DESeq2 have become a widely used method of performing differential abundance testing in microbiome analyses due to their ability to handle zero-inflated datasets, high dispersion, and account for non-normal distributions. However, a drawback of DESeq2 is that it does not allow for nested designs or designs with random effects. Thus, in our case, plant species could not be included the model testing for differential abundance because all other comparisons are linear combinations of this plant species factor – i.e., the comparisons between symbiotic groups are contrasts of the factor plant species. *P*-values were adjusted for multiple testing using the False Discovery Rate (FDR) method [106], and OTUs with an adjusted *p*-value < 0.05 and a minimal log_2_ fold-change (FC) of 1 were considered to be differentially abundant. As such, differentially abundant OTUs comprise OTUs that were significantly differentially abundant between two symbiosis groups. We then defined the symbiotype-specific OTUs (ss*-*OTUs) as the set of OTUs that were significantly more abundant in one symbiotype group compared to both other groups (intersect of two comparisons). For example, ss-OTUs for group *AR* were significantly more abundant in group *AR* in the comparison *AR* vs. *N* and *AR* vs. *A*. The sequences of the bacterial and fungal ss-OTUs were aligned with MUSCLE [107] and visualized in phylogenetic trees using the Interactive Tree of Life tool [108].

### Enrichment and depletion of microbial taxa

To test for enrichment or depletion of microbial taxa occurrences in the communities of OTUs with significant differences between the different symbiotype groups and the ss-OTUs, we constructed a contingency table for each taxon from the phylum to the family level with the within/outside taxon counts for the set of OTUs being considered and all filtered OTUs in the dataset and tested for significance with Fisher’s exact test. Expected counts for each contingency table were calculated as: *number of* significant OTUs * overall number of OTUs in taxon / total number of OTUs. P-values were FDR corrected for multiple testing, and taxonomic groups with an adjusted *p*-value < 0.05 were considered to be significantly enriched or depleted.

## Electronic supplementary material

Below is the link to the electronic supplementary material.

**Additional file 1**: A PDF containing supplementary methods, results, references, figures and tables. The SUPPLEMENTARY METHODS contain the details about the plant growth experiment, sample collection and DNA extraction, PCR setup, library preparation and sequencing, and bioinformatics and statistical analyses. The SUPPLEMENTARY RESULTS describe differences in root microbiome composition between the plant species based on the analysis of the data with the primary symbionts included. SUPPLEMENTARY FIGURES: **Figure S1** – Flow diagram of analysis steps. **Figure S2** – CAP ordinations of the dataset containing the primary symbionts. **Figure S3** –Unconstrained PCoA ordination of the dataset containing the primary symbionts. **Figure S4** – Percentage AMF root colonization of plant species from groups *A* and *AR. ***Figure S5 –** Rarefaction curves for datasets with and without the primary symbionts. **Figure S6 –** Bacterial and fungal OTU richness, effective richness, and Pielou’s evenness for and the individual plant species for the dataset with the primary symbionts removed. **Figure S7** – Unconstrained PCoA ordination of the dataset with the primary symbionts removed. SUPPLEMENTARY TABLES: **Table S1** – List of plant species used in the study. **Table S2** - Percentages of primary symbiont bacterial and fungal sequences removed per plant species. **Table S3** - Taxa occurrences at the phylum and class level of the bacterial and fungal OTU communities for datasets containing the primary symbionts and with the primary symbionts removed. **Table S4** - PERMANOVA table for the effects of plant species and symbiotic groups on community composition of bacterial and fungal OTU communities for the dataset containing the primary symbionts. **Table S5** - ANOVA table testing for differences in primary symbiont OTU relative abundance by symbiotype group. **Table S6** - ANOVA table testing for the effects of plant species and symbiotic groups on bacterial and fungal species richness, effective species richness, and Pielou’s evenness for the dataset with primary symbionts removed. **Table S7** – Number of significantly different bacterial and fungal OTUs between the different groupings of symbiotic status of plants for the dataset with primary symbionts removed. **Table S8** - Symbiotic group enriched bacterial and fungal OTUs for the three different symbiosis groups with the primary symbiotic OTUs removed.

**Additional file 2**: XLSX table with sample ID, barcode assignments, and primer sequences for each sample sequenced in both 16S rRNA and ITS MiSeq libraries.

**Additional file 3**: TXT file of all bacteria OTU representative sequences.

**Additional file 4**: TXT file of all fungal OTU representative sequences.

**Additional file 5**: Table of filtered bacteria OTU counts with taxonomy assignments in each sequenced sample.

**Additional file 6**: Table of filtered fungal OTU counts with taxonomy assignments in each sequenced sample.

**Additional file 7**: XLSX file of bacterial and fungal OTUs considered primary symbionts that were removed for some portions of the analysis and OTU counts of nodule samples used to define the bacteria primary symbionts.

**Additional file 8**: XLSX file of mean abundance (DESeq2 transformed counts) by plant species and symbiotype group, taxonomy assignment, symbiotype enrichment group, and primary symbiont status of the bacterial and fungal symbiotype specific OTUs.

## Data Availability

The raw sequencing is available from the European Nucleotide Archive under accession number PRJEB52032. R scripts used to perform the statistical analysis are available from: https://github.com/MWSchmid/Hartman-et-al.-2022.
