# Supplementary material for: A symbiotic footprint in the plant root microbiome
Source: Environ Microbiome. 2023 Jul 31;18:65. doi: 10.1186/s40793-023-00521-w (PMC10391997; doi:10.1186/s40793-023-00521-w)
Supplement: Supplementary file 1 — Additional file 1: A PDF containing supplementary methods, results, references, figures and tables. The SUPPLEMENTARY METHODS contain the details about the plant growth experiment, sample collection and DNA extraction, PCR setup, library preparation and sequencing, and bioinformatics and statistical analyses. The SUPPLEMENTARY RESULTS describe differences in root microbiome composition between the plant species based on the analysis of the data with the primary symbionts included. SUPPLEMENTARY FIGURES: Figure S1 – Flow diagram of analysis steps. Figure S2 – CAP ordinations of the dataset containing the primary symbionts. Figure S3 –Unconstrained PCoA ordination of the dataset containing the primary symbionts. Figure S4 – Percentage AMF root colonization of plant species from groups A and AR. Figure S5 – Rarefaction curves for datasets with and without the primary symbionts. Figure S6 – Bacterial and fungal OTU richness, effective richness, and Pielou’s evenness for and the individual plant species for the dataset with the primary symbionts removed. Figure S7 – Unconstrained PCoA ordination of the dataset with the primary symbionts removed. SUPPLEMENTARY TABLES: Table S1 – List of plant species used in the study. Table S2 - Percentages of primary symbiont bacterial and fungal sequences removed per plant species. Table S3 - Taxa occurrences at the phylum and class level of the bacterial and fungal OTU communities for datasets containing the primary symbionts and with the primary symbionts removed. Table S4 - PERMANOVA table for the effects of plant species and symbiotic groups on community composition of bacterial and fungal OTU communities for the dataset containing the primary symbionts. Table S5 - ANOVA table testing for differences in primary symbiont OTU relative abundance by symbiotype group. Table S6 - ANOVA table testing for the effects of plant species and symbiotic groups on bacterial and fungal species richness, effective species richness, and Pielou’s eve [file 40793_2023_521_MOESM1_ESM.pdf]

**Additional file 1:**

# A symbiotic footprint in the plant root microbiome

Kyle Hartman, Marc W. Schmid, Natacha Bodenhausen, S. Franz Bender, Alain Y. Held, Klaus Schlaeppi, Marcel G.A. van der Heijden

**This file includes:**

Supplementary Methods
Supplementary Results
Supplementary References
Figures S1 to S7
Tables S1 to S8

## Supplementary Methods

### PLANT GROWTH

Soil was collected from a grassland field site near Zürich, Switzerland (47°26’20’’N 8°31’40’’E), sieved to 2 mm, and stored at 4°C until use. Soil was mixed 1:1 by volume with sterilized quartz-sand and analyzed for chemical properties at the Labor für Boden-und Umweltanalytik (Eric Schweizer AG, Thun, Switzerland): pH 6.8, 7/13/78% (clay/silt/sand) and 7.8/1.05/ 1.07 mg $kg^{-1}$ (water-extractable N/P/K). The rationale for mixing sand into the field soil was to reduce soil nutrient levels and thereby to foster symbiotic interactions. 17 different plant species from five families were selected for this study (Table S1). The seeds of *Lotus japonicus* were sandpapered to improve germination. Because of inherent differences in root length and volume between the different plant species, we used two pot sizes in the growth experiment. Lupin, maize, wheat, tomato, tobacco and pea were sown in “large” pots (ø 8cm h 30cm) to allow for greater root length, while all others were sown in “small” pots (ø 10cm h 10cm). Seeds were sown directly in the sand/soil mixture and pots were then placed in a cold chamber for three days for stratification. To correct for potential differences in the seed microbiome among plant species, a seed slurry was prepared by grinding seeds of each species with an autoclaved mortar and pestle and resuspending in 2ml phosphate-buffered saline solution. 1ml of the slurry of each species was combined and further diluted twice. 200µl of this solution was applied to each pot. During the first few weeks of growth, extra plants were thinned out to have one plant per pot. Plants were fertilized over the course of the experiment with a low P (0.3mM $KH_2PO_4$) nutrient solution following Bodenhausen *et al*., [1].

### SAMPLE COLLECTION AND DNA EXTRACTION

Plants were harvested after 10 weeks, and the root-associated microbiota was sampled following the protocol in Bodenhausen *et al*., [1]. Briefly, the loosely attached soil was shaken from the roots, and the roots were washed three times in PBS buffer and stored at -80°C until DNA extraction. For species forming a known association with AMF, a subsample of roots was stored in 50% ethanol until root staining for microscopy. These samples were subsequently stained with pen ink, mounted on a microscope slide, and examined on a light microscope for percentage of AMF colonization using the

magnified intersection method with one hundred intersections per sample [2]. For legume species (Table S1), we prepared additional nodule samples by removing some of the nodules that formed on the roots with a sterile scalpel and stored them separately at -80°C. The separate nodule data was used to determine the nodule-specific OTUs to define the primary symbiont rhizobia species.

For DNA extraction, roots and separate nodule samples were lyophilized, placed in 2ml centrifuge tubes with one metal bead and ground to a fine powder for 2 min at 25 Hz using a Retsch TissueLyser (Retsch, Haan, Germany). DNA was extracted from the samples using the NucleoSpin 96 Soil kit (Macherey-Nagel, Düren, Germany) following the manufacturer's instructions and eluted twice in 50µL of the supplied elution buffer. DNA was subsequently quantified using a Quant-iT Picogreen dsDNA Assay Kit (Invitrogen, Eugene, OR USA) on a Varian Cary Eclipse fluorescence spectrometer (Agilent Technologies, Santa Clara, CA USA) and diluted to 1ng/µl.

### PCR AND SEQUENCING LIBRARY PREPARATION

The 16S rRNA gene amplicon library was generated using the PCR primers 799F [3] and 1193R [4], and the ITS amplicon library was generated using the PCR primers ITS1F [5] and ITS2 [6]. The primers were extended at the 5'end with an error-tolerant 8-mer barcode for multiplexed library sequencing selected from Faircloth and Glenn [7] (Additional File 2). Each 20µL PCR reaction contained: 8µL 5PRIME Hot Master Mix, 0.3% BSA, 200nM each primer, 3ng (16S) or 5ng (ITS) of DNA template, and the remaining volume sterile distilled water. PCR reactions were performed on an iCycler instrument (BioRad, Hercules, CA, USA) in triplicates and pooled together before inspecting each sample on an agarose gel for correct size and absence of contamination in non-template reactions. The ITS reactions were then purified with the NucleoSpin Gel and PCR Clean up Kit (Machery-Nagel, Düren, Germany) according to the manufacturer's instructions. The entire volume of the 16S rRNA gene reactions were loaded into an agarose gel to separate the 16S rRNA gene band (ca. ~450 bp) from the ~800 bp mitochondria product (also produced by the 16S primers) by gel electrophoresis. The band corresponding to the amplified 16S rRNA gene amplicon was cut from the gel and purified with the gel kit above according to the manufacturer's instructions. The purified reactions were quantified using the same Picogreen assay described above and pooled in equal amounts (15ng/sample for 16S, 30ng/sample

for ITS) to obtain a 16S and an ITS library. Subsequently, the volume of each library was reduced to ~200µL using a CentriVap centrifugal vacuum concentrator (Labconco Corp., Kansas City, MO, USA). Both libraries were then purified and concentrated with the Agencourt AMPure XP kit (Beckman Coulter, Brea, CA, USA), eluted twice in 50µL sterile water, and quantified with a Qubit dsDNA HS assay on a Qubit 2.0 fluorometer (Invitrogen, Carlsbad, CA, USA). The libraries were then combined and concentrated once more with AMPure and eluted twice in 75µL sterile water for MiSeq library preparation.

The MiSeq libraries were prepared at the Functional Genomics Center Zurich (www.fgcz.ch) with the NEBNext DNA library Ultra kit (New England Biolabs, Ipswich, MA, USA). After end-repairing and polyadenylating the amplicons, NEBNext Adaptors were ligated. The ligated samples were run on a 2% agarose gel and the desired fragment length was excised (50 bp ± the target fragment length). DNA from the gel was purified with MinElute Gel Extraction Kit (Qiagen, Hilden, Germany). Fragments containing NEBNext adapters on both ends were selectively enriched with PCR using four cycles. Quality and quantity of the enriched libraries were validated using Qubit (1.0) Fluorometer and Tapestation (Agilent Technologies, Santa Clara, CA, USA). The libraries were normalized to 4 nM in Tris-Cl 10 mM, pH 8.5, with 0.1% Tween 20. The libraries were sequenced at the FGCZ on the Illumina MiSeq Personal Sequencer (Illumina, San Diego, CA, U.S.A.) using a 600 cycle v3 Sequencing kit (Cat. no. MS-102-3003), in paired-end 2× 300 bp mode.

### Bioinformatics and sequence processing

Operational taxonomic units (OTUs) were generated with UPARSE [8] (usearch v10.0.024). Following removal of sequencing adapters and low-quality bases with fastp [9] (v0.20.0), paired-end reads were merged with usearch (parameters -fastq_mergepairs -fastq_maxdiffs 25). Merged reads were then truncated up to the 16S/ITS primer sequences (16S forward: AACMGGATTAGATACCCKG, 16S reverse: ACGTCATCCCCACCTTCC, ITS forward: CTTGGTCATTTAGAGGAAGTAA, ITS reverse: GCTGCGTTCTTCATCGATGC) and filtered for the presence of both primer sequences with a custom python script allowing up to 2 mismatches per primer. Primers were then clipped. Merged reads were further quality-filtered with usearch (parameters -fastq_filter -fastq_maxee 1). For the ITS

sequence data, the highly variable subregion ITS1 was extracted with ITSx v1.1.1 [10] (parameters -t funghi –reset T –preserve T –save_regions ITS1). Duplicated sequences were collapsed with fqtrim [11] (v0.9.7) and denoised with usearch (parameter -unoise3). The fungal ITS1 data were then filtered for chimeras using the UNITE database [12] (v8.3) and usearch (with the parameters -uchime_ref -strand plus -mode balanced). Remaining sequences were sorted according to their length (required for usearch -cluster_smallmem) and clustered with a minimal identity threshold of 99 % using usearch (parameters -sortbylength -minseqlength 64 for sorting and -cluster_smallmem -id 0.99 for clustering). Finally, we obtained 6,052 bacterial and 706 fungal OTU sequences (Fig. S1, Additional files 3 & 4).

16S-OTU sequences were annotated with the taxonomy data available from the Ribosomal Database Project [13] (bacterial sequences, v16) and the ITS OTU sequences with a combination of the PLANiTS database [14] (status March 2020) and UNITE v8.3 with the usearch command *sintax* [15] (v10.0.240; parameters -sintax -strand both -sintax_cutoff 0.8). Any OTU sequences classified as Viridiplantae were removed. Fungal ITS-OTUs were further annotated with functional categories using FUNGuild [16] (v1.1). OTU abundances were obtained by counting the number of sequences (merged and filtered) matching to the OTU sequences (parameters -otutab -strand plus). To avoid sequencing artifacts, OTU sequences with less than 30 (16S) or 10 (ITS) counts in total or with counts in less than three samples were removed from all further analyses (4,327 bacterial and 488 fungal OTUs remained after this filter, Fig. S1, Additional files 5 & 6).

To identify the primary symbionts (Rhizobia and AMF) in the root microbiomes, we marked 16S-OTUs annotated as *Rhizobium*, *Mesorhizobium*, *Bradyrhizobium* or *Azorhizobium* with at least 10 raw reads across all nodule samples of legume plants as nodule-specific symbiotic OTUs (52 OTUs). Likewise, ITS-OTUs annotated as Glomeromycota in root samples of all plant species were marked as specific AMF OTUs (127 OTUs). We refer to these nodule- and AMF-specific OTUs collectively as ‘primary symbionts’, and for specific analyses – i.e., to test whether changes in the root microbiome are also detected without these taxa – these OTUs were removed from their respective datasets (Fig. S1, Table S2, Additional file 7).

### ANALYSIS OF DIVERSITY, BETWEEN SAMPLE DISTANCES, AND COMMUNITY STRUCTURE

Counts of the filtered OTU sequences were rarefied to the sample with the lowest number of counts in the dataset (22,485 bacteria/3,387 fungi with primary symbionts and 10,882 bacteria/1,090 fungi without the primary symbionts; Fig. S5).

Unconstrained principal coordinates analysis (PCoA) and constrained analysis of principal coordinates (CAP) analyses were performed on Bray-Curtis dissimilarities calculated from rarefied OTU abundances. In the CAP model, *seqDepth* (number of original, unrarefied sequences per sample) and *pot* (type of pot used) were considered as conditioning factors and *AMF* (presence/absence of AMF), *Rhizobia* (presence/absence of rhizobia), and *Species_name* (plant species) as constraining factors (*OTU_counts ~ Condition(seqDepth) + Condition(pot) + AMF + Rhizobia + Species_name)*.

We assessed the impact of the symbiotic groups on community structure with a permutational multivariate ANOVA (PERMANOVA) in R with the function *adonis* from the R package *vegan* [17] with 999 permutations. This was performed on Bray-Curtis dissimilarities calculated from rarefied datasets with and without the primary symbionts. For assessing the effects of the symbiotic groups on both the diversity indices and community dissimilarities, we used the same model detailed below.

## ANOVA AND PERMANOVA MODELS

The structure of all (PERM)ANOVA models followed general design principles (see Schmid *et al.,* [18] for a detailed discussion of this approach). For all models, factors were fitted sequentially (type I sum of squares). Significance tests were based on *F*-tests calculated manually using appropriate error terms and denominator degrees of freedom. To correct for differences in sequencing depth and to account for differences between the two different pot types used in the experiment, the factors *sequencing depth* and *pot* were fitted first. Because the symbiotic group characterized by no symbiosis with AMF but with rhizobia consisted of Lupin only (*R*), we next fitted a factor for the plant species being lupin or not (*isLupin*). We then fitted a factor *symbiotic group*, representing the symbiotic type of the remaining plant species, and consisting of the levels *N* (*N*o symbiosis with AMF or rhizobia), *A* (symbiosis with *A*MF, but not with rhizobia), and *AR* (symbiosis with *A*MF and *R*hizobia). To compare the groups to each other, the factor *symbiotic group* was split into three different sets of contrasts. In each set, one group was first compared to the others, and the remaining two groups were then compared

to each other, so that the model was run three times to obtain the results for all three pairwise comparisons. Next, we fit additional model terms to account for the effect of the different plant families within each symbiotic group. However, only group *A* (AMF only; Poaceae and Solanaceae) and group *N* (neither symbiont; Amaranthaceae and Brassicaceae) could be included as group *R* and *AR* did not contain multiple families. Finally, we included three additional terms to test for differences between the individual plant species that comprise symbiotype groups *A*, *AR*, and *N.* We chose this modeling technique over other possible methods (e.g., phylogenetic least squares regression) because our symbiotype groups were not equally distributed across the phylogeny of the profiled plant species.

### PHYLOGENETIC ANALYSES BETWEEN PLANT SPECIES AND COMMUNITY COMPOSITION

The midpoint rooted phylogenetic tree of the different plant species was generated using the maximum likelihood method with the Jukes-Cantor model and 1000 bootstrap replications using the R package *phangorn* [19] (v2.7.1).

To create the plant phylogenetic matrix to assess the correlation between plant phylogenetic similarity and a Bray-Curtis dissimilarity matrix of the bacterial or fungal communities, we took the *rcbL* gene sequences for each plant species and duplicated the entries to account for the replicate samples within each species (91 total sequences). We then calculated a pairwise evolutionary distance matrix of the plant species using the Jukes-Cantor model with the R package *ape* [20] (v5.5).

## Supplementary Results

### PRESENCE OF PRIMARY SYMBIONTS IN MICROBIOME DRIVES DIFFERENCES IN COMMUNITY COMPOSITION

We evaluated the overall differences in community composition between microbiomes of the different symbiotic groups by conducting CAP ordinations on Bray-Curtis dissimilarities calculated using the rarified b-OTU and f-OTU abundances of the dataset with the primary symbionts included and the factors presence/absence of rhizobia and presence/absence of AMF as explanatory terms (Fig. S1, Fig. S2). Samples in the b-OTU community were mostly separated between legumes and non-legume species, whereas the differences in the f-OTU community were less apparent (Fig. S2). The

primary CAP axes explained 25.5% and 32.6% of the overall variances in the b-OTU and f-OTU communities, respectively (Fig. S2, see also Table S4). Unconstrained PCoA ordinations revealed mostly similar patterns, with Axis 1 separating legumes and non-legume on in the b-OTU community and group *N* species clearly separating from the species of the other groups on Axis 1 in the f-OTU community (Fig. S3).

### RELATIVE EFFECT SIZES OF SYMBIOTYPE AND PLANT SPECIES DRIVING MICROBIOME COMPOSITION

Next, we quantified the effect sizes of the different symbiotic groups on b-OTU and f-OTU community composition using PERMANOVA. This analysis corroborated the symbiotypes, with a significant symbiotic group effect explaining 10.5% of variance (in terms of % SS) in the b-OTUs and 14.9% in the f-OTUs containing the primary symbionts (Table S4). However, differences between families in groups *A* and *N* and between plant species from groups *A*, *AR*, and *N* was the strongest determinant of b-OTU community composition, explaining a total of 6.4% and 23.5% of overall variance, respectively (Table S4). In the f-OTU community, significant differences between families in groups A and N and species in groups A and AR explained a combined 15% and 16.8% of variance respectively.

In short, plant family and species identity are strong and significant drivers of differences in root microbiome composition. However, as expected, the presence of rhizobia and AMF also presents a significant driver community composition.

## Supplementary References

1. Bodenhausen N, Somerville V, Desirò A, Walser J-C, Borghi L, van der Heijden MGA, et al. Petunia- and Arabidopsis-Specific Root Microbiota Responses to Phosphate Supplementation. Phytobiomes J. 2019;3:112–24. doi:10.1094/pbiomes-12-18-0057-r.

2. McGonigle T, Miller M, Evans D, Fairchild G, Swan J. A new method which gives an objective measure of colonization of roots by vesicular-arbuscular mycorrhizal fungi. New Phytol. 1990;115:495.

3. Chelius MK, Triplett EW. The diversity of archaea and bacteria in association with the roots of Zea mays L. Microb Ecol. 2001;41:252–63. doi:10.1007/s002480000087.

4. Bodenhausen N, Horton MW, Bergelson J. Bacterial Communities Associated with the Leaves and the Roots of Arabidopsis thaliana. PLoS One. 2013;8. doi:10.1371/journal.pone.0056329.

5. Gardes M, Bruns TD. ITS primers with enhanced specificity for basidiomycetes - application to the identification of mycorrhizae and rusts. Mol Ecol. 1993;2:113–8. doi:10.1111/j.1365-294X.1993.tb00005.x.

6. White T, Bruns T, Lee S, Taylor J. Amplification and direct sequencing of fungal ribosomal RNA genes for phylogenetics. PCR Protocols: A Guide to Methods and Applications. 1990.

7. Faircloth BC, Glenn TC. Not all sequence tags are created equal: Designing and validating sequence identification tags robust to indels. PLoS One. 2012;7. doi:10.1371/journal.pone.0042543.

8. Edgar RC. UPARSE: Highly accurate OTU sequences from microbial amplicon reads. Nat Methods. 2013;10:996–8. doi:10.1038/nmeth.2604.

9. Chen S, Zhou Y, Chen Y, Gu J. Fastp: An ultra-fast all-in-one FASTQ preprocessor. Bioinformatics. 2018;34:i884–90. doi:10.1093/bioinformatics/bty560.

10. Bengtsson-Palme J, Ryberg M, Hartmann M, Branco S, Wang Z, Godhe A, et al. Improved software detection and extraction of ITS1 and ITS2 from ribosomal ITS sequences of fungi and other eukaryotes for analysis of environmental sequencing data. Methods Ecol Evol. 2013;4:914–9. doi:10.1111/2041-210X.12073.

11. Pertea G. fqtrim 0.9.7. 2015.

12. Nilsson RH, Larsson KH, Taylor AFS, Bengtsson-Palme J, Jeppesen TS, Schigel D, et al. The UNITE database for molecular identification of fungi: Handling dark taxa and parallel taxonomic classifications. Nucleic Acids Res. 2019;47:D259–64. doi:10.1093/nar/gky1022.

13. Cole JR, Wang Q, Fish JA, Chai B, McGarrell DM, Sun Y, et al. Ribosomal Database Project: Data and tools for high throughput rRNA analysis. Nucleic Acids Res. 2014;42:633–42. doi:10.1093/nar/gkt1244.

14. Banchi E, Ametrano CG, Greco S, Stanković D, Muggia L, Pallavicini A. PLANiTS: A curated sequence reference dataset for plant ITS DNA metabarcoding. Database. 2020;2020. doi:10.1093/database/baz155.

15. Edgar R. SINTAX: a simple non-Bayesian taxonomy classifier for 16S and ITS sequences. bioRxiv. 2016;074161. doi:10.1101/074161.

16. Nguyen NH, Song Z, Bates ST, Branco S, Tedersoo L, Menke J, et al. FUNGuild: An open annotation tool for parsing fungal community datasets by ecological guild. Fungal Ecol. 2016;20:241–8. doi:10.1016/j.funeco.2015.06.006.

17. Oksanen J, Blanchet FG, Friendly M, Kindt R, Legendre P, McGlinn D, et al. vegan: Community Ecology Package. 2017.

18. Schmid B, Baruffol M, Wang Z, Niklaus PA. A guide to analyzing biodiversity experiments. Journal of Plant Ecology. 2017;10:91–110. doi:10.1093/jpe/rtw107.

19. Schliep KP. phangorn: Phylogenetic analysis in R. Bioinformatics. 2011;27:592–3. doi:10.1093/bioinformatics/btq706.

20. Paradis E, Schliep K. Ape 5.0: An environment for modern phylogenetics and evolutionary analyses in R. Bioinformatics. 2019;35:526–8. doi:10.1093/bioinformatics/bty633.

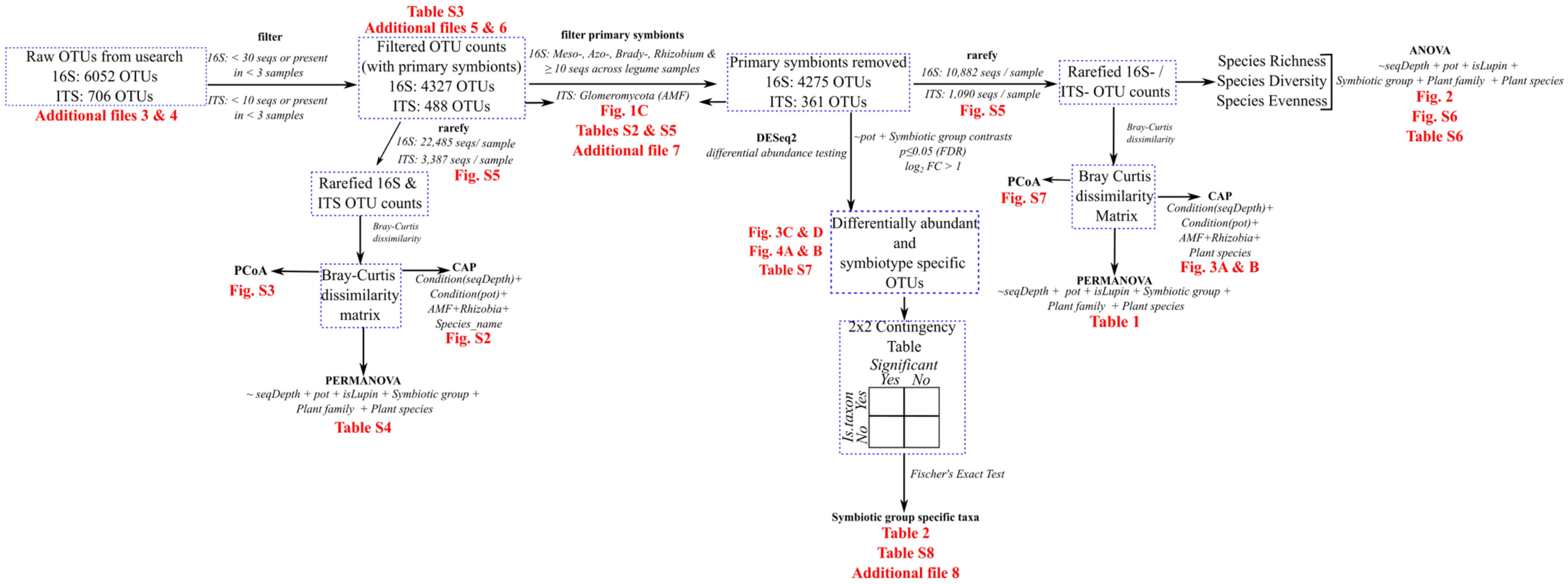


**Figure S1**: Schematic flow diagram of analysis steps. Boxes outlined in blue represent input datasets for the different analyses performed. The figures, tables, or additional files generated as the output from each step are indicated in red.

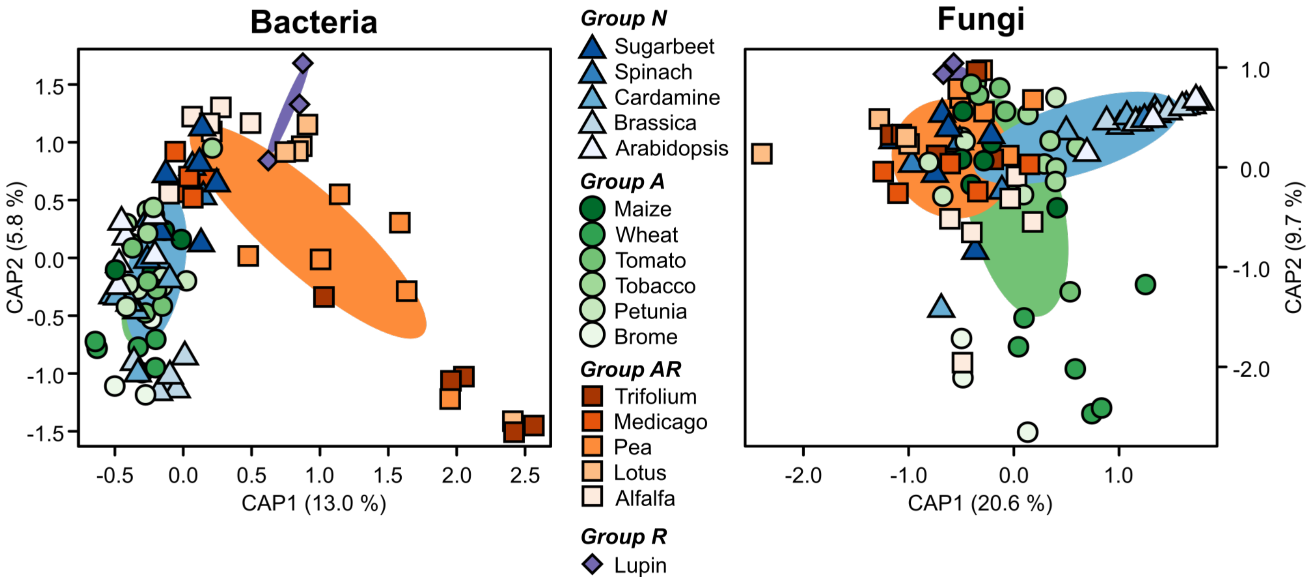


**Figure S2:** Constrained analysis of principal coordinates (CAP) on Bray Curtis dissimilarities of all samples calculated on the dataset containing the primary symbionts. Data points show individual replicates of the 17 different plant species. Ellipses represent the 95% confidence interval of the group centroids. The percentage of total variance explained by each CAP axis is shown in parentheses.

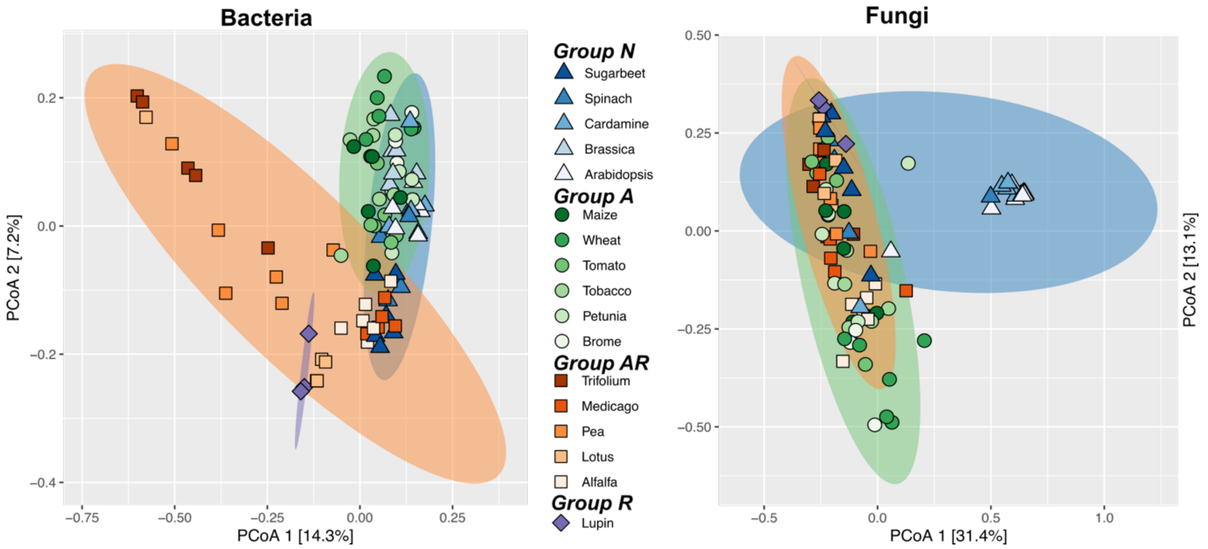


**Figure S3:** Unconstrained analysis of principal coordinates (PCoA) on Bray Curtis dissimilarities of all samples calculated on the dataset containing the primary symbionts. Data points show individual replicates of the 17 different plant species. Ellipses represent the 95% confidence interval of the group centroids. The percentage of total variance explained by each axis is shown in parentheses

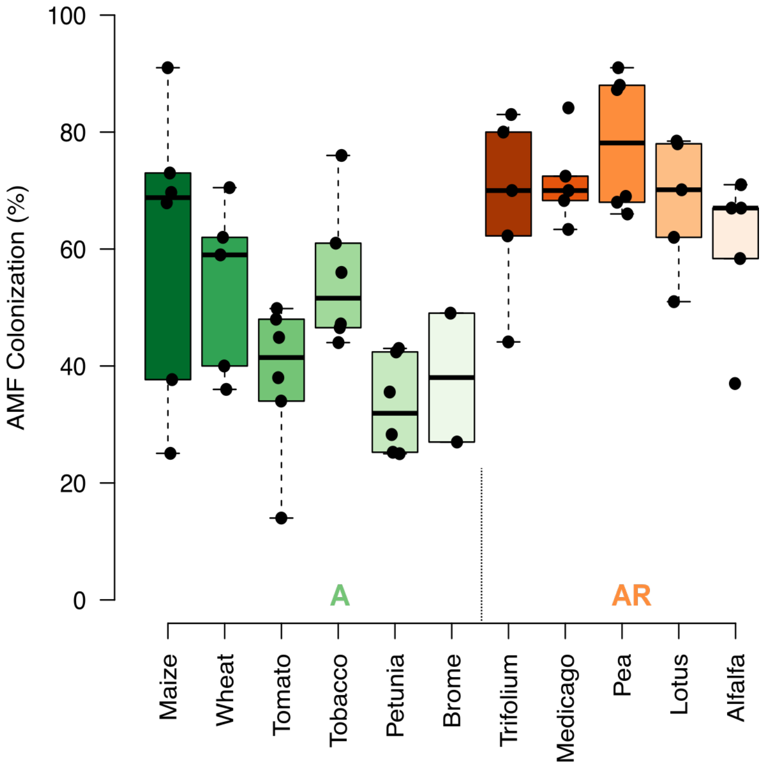


**Figure S4**: Microscopic determination of AMF root colonization of plant species from groups *A* and *AR*. Root colonization was assessed by the intersection method on 100 intersections per sample.

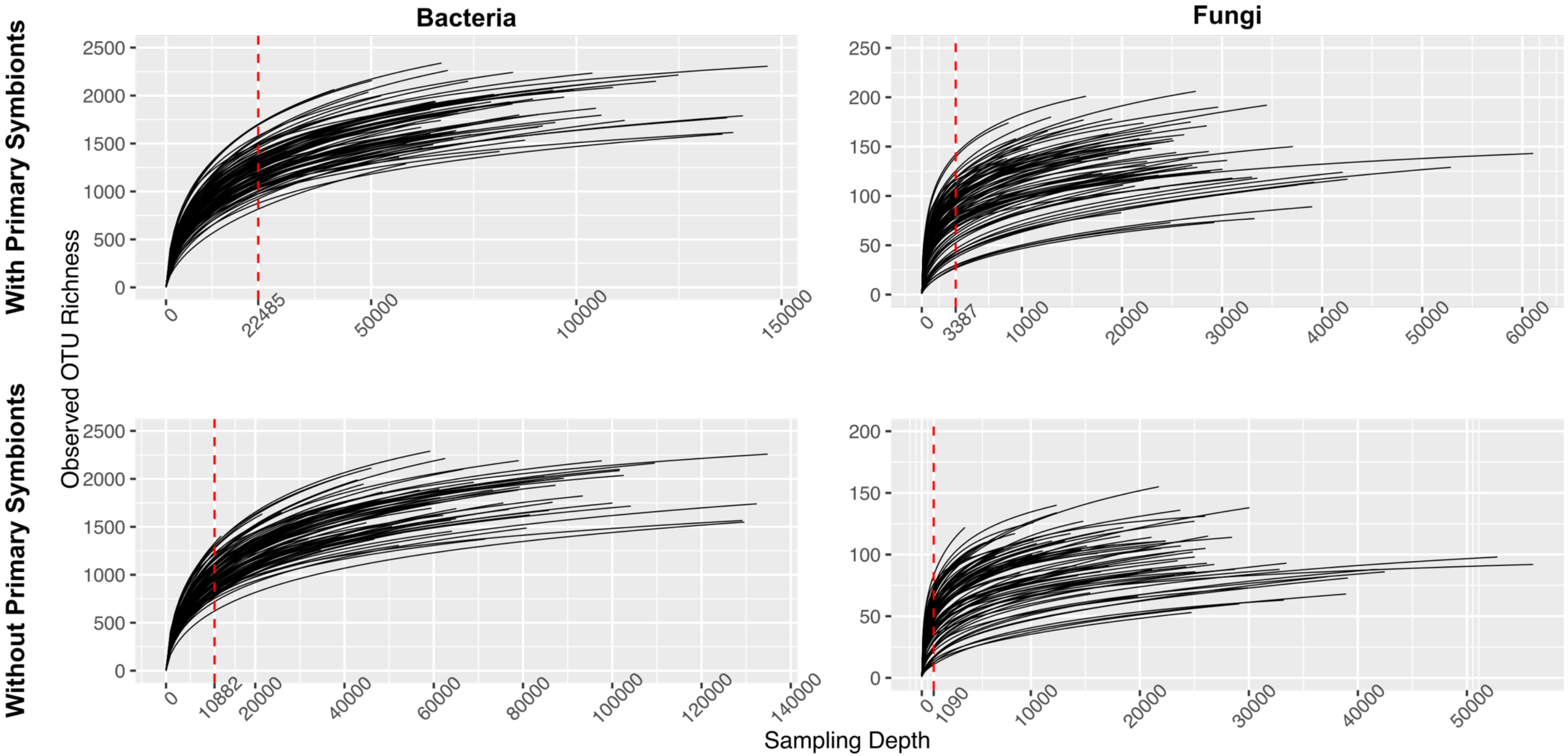


**Figure S5**: Rarefaction curves of bacterial and fungal data sets containing the primary symbionts and with the primary symbionts removed. The red dashed lines indicate the rarefaction depth of each community marked on the x-axis

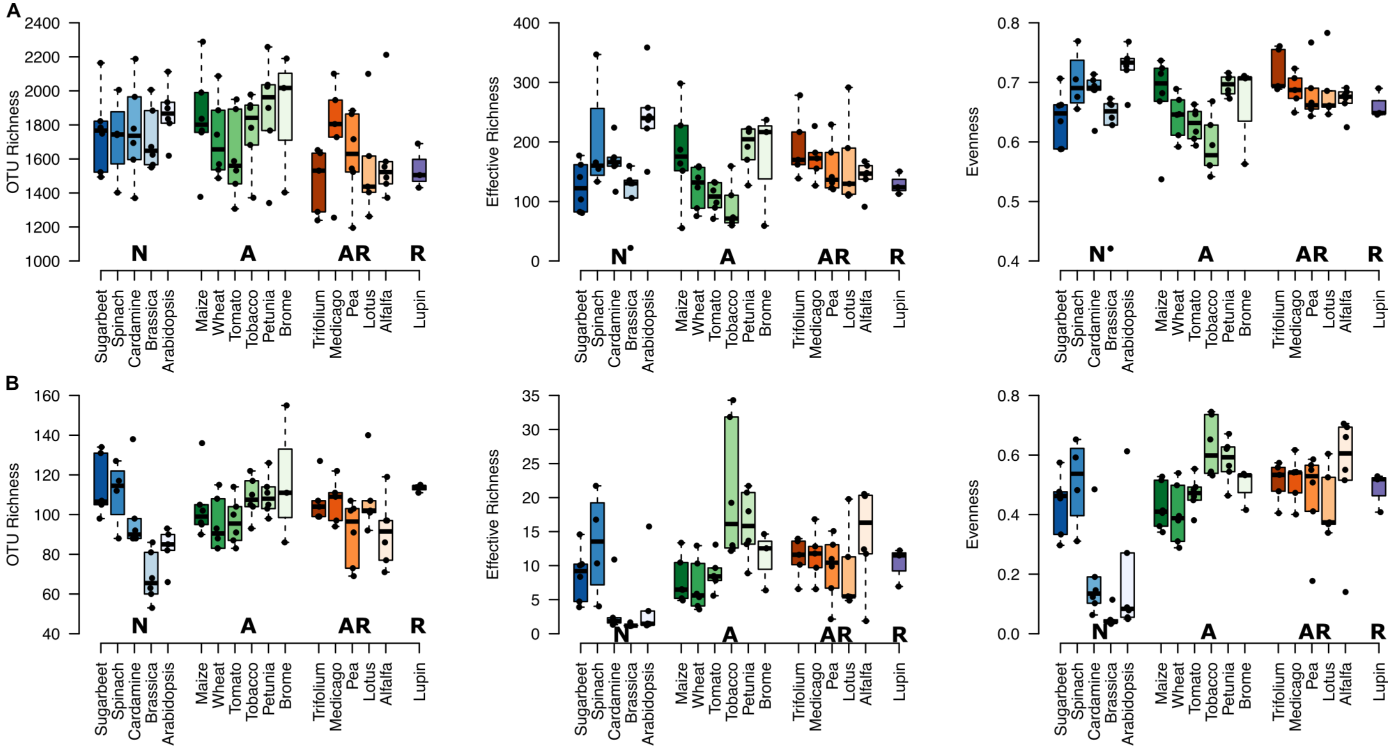


**Figure S6:** Bacterial (**A**) and fungal (**B**) OTU richness, effective richness, and Pielou's evenness for the individual plant species for the dataset with the primary symbionts removed. Symbiotype groups are indicated as species without AMF and without rhizobia (*N*), plant species with AMF but without rhizobia (*A*), plant species with AMF and rhizobia (*AR*). Note that Lupin is the only representative species for group *R* which associates with rhizobia but not AMF. The bottom and top of the boxes correspond to the lower and upper quartiles and the center line marks the median. Whiskers extend to the lowest/highest values unless these values are lower/higher than the first/third quartile minus/plus 1.5 times the inner quartile range, which equals the third minus the first quartile.

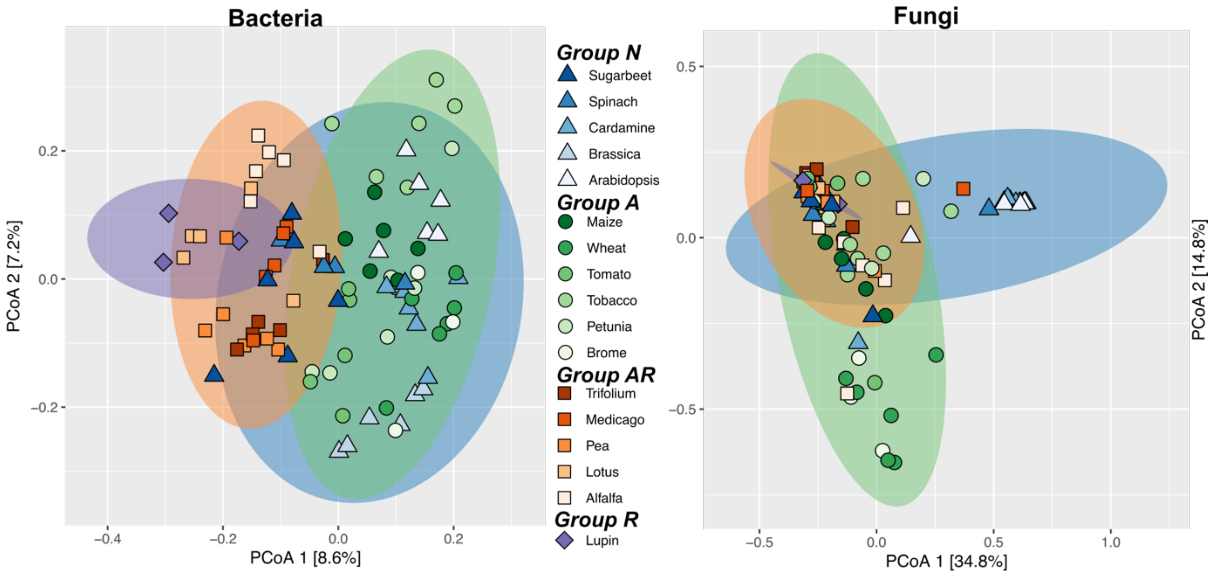


**Figure S7:** Unconstrained analysis of principal coordinates (PCoA) on Bray Curtis dissimilarities of all samples calculated on the dataset with the primary symbionts removed. Data points show individual replicates of the 17 different plant species. Ellipses represent the 95% confidence interval of the group centroids. The percentage of total variance explained by each axis is shown in parentheses.

**Table S1**: List of plant species used in this study. All replicate root samples from plant species in the Fabaceae were extracted with any nodules present. However, cluster root structures were removed from Lupin replicates. The Genbank accession number of the *rbcL* gene for each species was used to produce the phylogenetic tree in Figure 1.

| Plant | Species | Family | AMF | Rhizobia | Symbiotic Group | Accession / Variety | Number of Replicates | Genbank Accession |
|---|---|---|---|---|---|---|---|---|
| Spinach | *Spinacia oleracea* | Amaranthaceae | No | No | N | Andromeda | 4 | NC_002202.1 |
| Sugar beet | *Beta vulgaris* | Amaranthaceae | No | No | N | Samuela | 6 | AY270065.1 |
| Bittercress | *Cardamine hirsuta* | Brassicaceae | No | No | N | Oxford | 6 | NC_049607.1 |
| Rapeseed | *Brassica napus* | Brassicaceae | No | No | N | Harry | 6 | NC_016734.1 |
| Thale cress | *Arabidopsis thaliana* | Brassicaceae | No | No | N | Col0 | 6 | NC_000932.1 |
| Brome | *Brachypodium distachyon* | Poaceae | Yes | No | A | Bd21 | 3 | NC_011032.1 |
| Maize | *Zea mays* | Poaceae | Yes | No | A | Fast-Flowering Mini-Maize (line A) | 6 | NC_001666.2 |
| Wheat | *Triticum aestivum* | Poaceae | Yes | No | A | Fiorina | 6 | NC_002762.1 |
| Petunia | *Petunia hybrida* | Solanaceae | Yes | No | A | V26 | 6 | NC_040178.1 |
| Tomato | *Solanum lycopersicum* | Solanaceae | Yes | No | A | Craigella | 6 | NC_007898.3 |
| Tobacco | *Nicotiana benthamiana* | Solanaceae | Yes | No | A | n/a | 6 | NC_001879.2 (*Nicotiana tabacum*) |
| Birdsfoot trefoil | *Lotus japonicus* | Fabaceae | Yes | Yes | AR | Gifu | 5 | NC_002694.1 |
| Barrelclover | *Medicago truncatula* | Fabaceae | Yes | Yes | AR | Jemalong A17 | 5 | NC_003119.8 |
| Pea | *Pisum sativum* | Fabaceae | Yes | Yes | AR | Enduro | 6 | NC_014057.1 |
| Red clover | *Trifolium pratense* | Fabaceae | Yes | Yes | AR | Milvus | 5 | NC_047412.1 |
| Alfalfa | *Medicago sativa* | Fabaceae | Yes | Yes | AR | Sanditi | 6 | NC_042841.1 |
| White lupin | *Lupinus albus* | Fabaceae | No | Yes | R | Amiga | 3 | NC_026681.1 |

**Table S2**: Percentages of symbiotic bacterial and fungal sequences removed per plant species. Bacterial OTUs annotated as *Rhizobium, Mesorhizobium, Bradyrhizobium* or *Azorhizobium* and with at least 10 reads across all nodule samples of legume plants were considered as nodule-specific symbiotic OTUs (52 OTUs); Fungal OTUs annotated as Glomeromycota in root samples were marked as specific AMF symbionts (127 OTUs).

| Plant | % Reads Removed (16S) | % Reads Removed (ITS) |
|---|---|---|
| Alfalfa | 14 | 46 |
| Arabidopsis | 11 | 3 |
| Brassica | 7 | 0 |
| Brome | 8 | 13 |
| Cardamine | 12 | 3 |
| Lotus | 30 | 14 |
| Lupin | 37 | 1 |
| Maize | 11 | 18 |
| Medicago | 13 | 46 |
| Pea | 36 | 18 |
| Petunia | 9 | 31 |
| Spinach | 9 | 2 |
| Sugarbeet | 13 | 1 |
| Tobacco | 8 | 64 |
| Tomato | 10 | 19 |
| Trifolium | 59 | 30 |
| Wheat | 8 | 6 |

**Table S3:** Taxa occurrences at the phylum and class level of the bacterial and fungal OTU communities for datasets containing the primary symbionts and with the primary symbionts removed. Values in the "RelAbund" column give the percentage of rarefied sequences of each dataset belonging to the indicated taxonomy.

| **Bacteria** | | | | |
|---|---|---|---|---|
| | ***with primary symbionts*** | | ***without primary symbionts*** | |
| Phylum | OTU Count | RelAbund | OTU Count | RelAbund |
| ***Cyanobacteria*** | 1 | <0.01 | 1 | <0.01 |
| ***Thaumarchaeota*** | 1 | <0.01 | 1 | <0.01 |
| ***Gemmatimonadetes*** | 3 | 0.01 | 3 | <0.01 |
| ***candidate_division_WPS-1*** | 5 | 0.01 | 5 | 0.01 |
| ***Nitrospirae*** | 5 | 0.02 | 5 | 0.05 |
| ***Parcubacteria*** | 6 | 0.01 | 6 | 0.03 |
| ***Spirochaetes*** | 9 | 0.29 | 9 | 0.37 |
| ***Chloroflexi*** | 10 | 0.12 | 10 | 0.24 |
| ***Deinococcus-Thermus*** | 10 | 0.08 | 10 | 0.06 |
| ***Armatimonadetes*** | 38 | 1.27 | 38 | 0.36 |
| ***Candidatus_Saccharibacteria*** | 46 | 0.53 | 46 | 0.27 |
| ***Planctomycetes*** | 72 | 0.6 | 72 | 0.8 |
| ***Chlamydiae*** | 77 | 0.65 | 77 | 0.56 |
| ***Verrucomicrobia*** | 96 | 0.59 | 96 | 1.92 |
| ***Acidobacteria*** | 160 | 1.14 | 160 | 1.38 |
| ***Firmicutes*** | 252 | 8.72 | 252 | 9.4 |
| ***Bacteroidetes*** | 303 | 5.92 | 303 | 4.73 |
| ***Actinobacteria*** | 655 | 20.98 | 655 | 12.03 |
| **unassigned** | 657 | 7.86 | 657 | 9.2 |
| ***Proteobacteria*** | 1921 | 51.2 | 1869 | 58.58 |
| **Total** | **4327** | | **4275** | |

| | ***with primary symbionts*** | | ***without primary symbionts*** | |
|---|---|---|---|---|
| Class | OTU Count | RelAbund | OTU Count | RelAbund |
| ***Acidobacteria_Gp22*** | 1 | <0.01 | 1 | <0.01 |
| ***Caldilineae*** | 1 | 0.01 | 1 | 0.01 |
| ***Chloroplast*** | 1 | <0.01 | 1 | <0.01 |
| ***Ktedonobacteria*** | 1 | 0.01 | 1 | <0.01 |
| ***Nitrososphaerales*** | 1 | <0.01 | 1 | <0.01 |
| ***Bacteroidia*** | 2 | <0.01 | 2 | 0.02 |
| ***Erysipelotrichia*** | 2 | 0.02 | 2 | 0.02 |
| ***Holophagae*** | 2 | 0.01 | 2 | <0.01 |
| ***Oligoflexia*** | 2 | 0.03 | 2 | 0.08 |
| ***Armatimonadetes_gp5*** | 2 | <0.01 | 2 | <0.01 |
| ***Parcubacteria_genera_incertae_sedis*** | 2 | <0.01 | 2 | 0.02 |
| ***Fimbriimonadia*** | 3 | <0.01 | 3 | 0.03 |
| ***Gemmatimonadetes*** | 3 | 0.01 | 3 | <0.01 |
| ***Verrucomicrobiae*** | 3 | <0.01 | 3 | <0.01 |
| ***Acidobacteria_Gp6*** | 4 | 0.02 | 4 | 0.01 |
| ***Acidobacteria_Gp7*** | 5 | 0.07 | 5 | 0.01 |
| ***Chloroflexia*** | 5 | 0.01 | 5 | 0.01 |
| ***Nitrospira*** | 5 | 0.02 | 5 | 0.05 |
| ***Acidobacteria_Gp2*** | 6 | 0.05 | 6 | 0.04 |
| ***Acidobacteria_Gp4*** | 8 | 0.02 | 8 | 0.06 |
| ***Spartobacteria*** | 9 | 0.04 | 9 | 0.34 |
| ***Spirochaetia*** | 9 | 0.29 | 9 | 0.37 |
| ***Acidobacteria_Gp5*** | 10 | 0.14 | 10 | 0.08 |
| ***Armatimonadia*** | 10 | 0.06 | 10 | 0.04 |
| ***Deinococci*** | 10 | 0.08 | 10 | 0.06 |
| ***Acidobacteria_Gp10*** | 13 | 0.05 | 13 | 0.23 |
| ***Saccharibacteria_genera_incertae_sedis*** | 28 | 0.08 | 28 | 0.11 |
| ***Acidobacteria_Gp1*** | 49 | 0.29 | 49 | 0.32 |
| ***Flavobacteriia*** | 50 | 3.12 | 50 | 1.17 |
| ***Clostridia*** | 54 | 1.27 | 54 | 1.69 |
| ***Acidobacteria_Gp3*** | 60 | 0.45 | 60 | 0.54 |
| ***Planctomycetia*** | 72 | 0.6 | 72 | 0.8 |
| ***Chlamydiia*** | 77 | 0.65 | 77 | 0.56 |
| ***Subdivision3*** | 80 | 0.53 | 80 | 1.58 |
| ***Cytophagia*** | 95 | 1 | 95 | 0.96 |
| ***Sphingobacteriia*** | 107 | 1.22 | 107 | 1.79 |
| ***Deltaproteobacteria*** | 149 | 6.09 | 149 | 2.54 |

| | | | | |
|---|---|---|---|---|
| ***Bacilli*** | 195 | 7.44 | 195 | 7.71 |
| ***Gammaproteobacteria*** | 244 | 6.77 | 244 | 5.24 |
| ***Betaproteobacteria*** | 438 | 10.95 | 438 | 13.54 |
| ***Actinobacteria*** | 647 | 20.86 | 647 | 11.96 |
| **unassigned** | 917 | 12.85 | 916 | 15.88 |
| ***Alphaproteobacteria*** | 945 | 24.87 | 894 | 32.12 |
| **Total** | **4327** | | **4275** | |
| | | | | |
| **Fungi** | | | | |
| | ***with primary symbionts*** | | ***without primary symbionts*** | |
| Phylum | OTU Count | RelAbund | OTU Count | RelAbund |
| ***Olpidiomycota*** | 1 | 19.58 | 1 | 21.47 |
| ***Zoopagomycota*** | 2 | <0.01 | 2 | 0.01 |
| ***Rozellomycota*** | 3 | 0.07 | 3 | 0.11 |
| ***Mortierellomycota*** | 7 | 0.68 | 7 | 0.99 |
| ***Chytridiomycota*** | 9 | 0.6 | 9 | 0.83 |
| ***Basidiomycota*** | 28 | 0.49 | 28 | 0.82 |
| ***Glomeromycota*** | 127 | 21.04 | - | - |
| ***Ascomycota*** | 132 | 17.98 | 132 | 24.47 |
| **unassigned** | 179 | 39.57 | 179 | 51.29 |
| **Total** | **488** | | **361** | |
| | | | | |
| | ***with primary symbionts*** | | ***without primary symbionts*** | |
| Class | OTU Count | RelAbund | OTU Count | RelAbund |
| ***Laboulbeniomycetes*** | 1 | <0.01 | 1 | <0.01 |
| ***Olpidiomycetes*** | 1 | 19.58 | 1 | 21.47 |
| ***Rozellomycotina_cls_Incertae_sedis*** | 1 | <0.01 | 1 | <0.01 |
| ***Wallemiomycetes*** | 1 | 0.01 | 1 | 0.03 |
| ***Zoopagomycetes*** | 1 | <0.01 | 1 | <0.01 |
| ***Chytridiomycetes*** | 2 | 0.55 | 2 | 0.74 |
| ***Rhizophlyctidomycetes*** | 2 | 0.01 | 2 | 0.02 |
| ***Rhizophydiomycetes*** | 2 | <0.01 | 2 | 0.01 |
| ***Malasseziomycetes*** | 3 | 0.02 | 3 | 0.04 |
| ***Microbotryomycetes*** | 4 | 0.01 | 4 | 0.01 |
| ***Orbiliomycetes*** | 6 | 0.05 | 6 | 0.08 |
| ***Agaricomycetes*** | 7 | 0.04 | 7 | 0.06 |
| ***Mortierellomycetes*** | 7 | 0.68 | 7 | 0.99 |
| ***Saccharomycetes*** | 7 | 0.01 | 7 | 0.03 |
| ***Leotiomycetes*** | 10 | 1.66 | 10 | 2.11 |
| ***Archaeosporomycetes*** | 13 | 0.03 | - | - |
| ***Eurotiomycetes*** | 13 | 2.74 | 13 | 4.04 |
| ***Paraglomeromycetes*** | 13 | 0.09 | - | - |
| ***Tremellomycetes*** | 13 | 0.41 | 13 | 0.68 |
| ***Dothideomycetes*** | 17 | 1.51 | 17 | 2.68 |
| ***Sordariomycetes*** | 62 | 7.67 | 62 | 9.8 |
| ***Glomeromycetes*** | 97 | 20.77 | - | - |
| **unassigned** | 205 | 44.15 | 201 | 57.21 |
| **Total** | **488** | | **361** | |

**Table S4:** Results of PERMANOVA testing for the effects of symbiotic groups, plant families, and plant species and on community composition (Bray-Curtis dissimilarities) of bacterial and fungal OTU communities for the dataset containing the primary symbionts. For the term “Symbiotic Group”, three different contrasts testing all possible combinations of factor levels of symbiotic groups against each other were performed (a, b, c).

| **Bacteria** | **df** | **F** | ***P*** | **%-SS** | **Fungi** | **F** | **P** | **%-SS** |
|---|---|---|---|---|---|---|---|---|
| seqDepth | 1 | 2.45 | ***0.001*** | 1.76 | seqDepth | 11.84 | ***0.001*** | 6.56 |
| Pot | 1 | 5.04 | ***0.001*** | 3.62 | Pot | 8.24 | ***0.001*** | 4.56 |
| isLupin | 1 | 3.63 | ***0.001*** | 2.6 | isLupin | 4.21 | ***0.001*** | 2.33 |
| Symbiotic Group | 2 | 7.29 | ***0.001*** | 10.45 | Symbiotic Group | 13.46 | ***0.001*** | 14.91 |
| *a) A vs others* | 1 | 6.41 | ***0.001*** | 4.6 | *a) A vs others* | 6.34 | ***0.001*** | 3.51 |
| *a) AR vs N* | 1 | 8.16 | ***0.001*** | 5.85 | *a) AR vs N* | 20.59 | ***0.001*** | 11.4 |
| *b) AR vs others* | 1 | 11.74 | ***0.001*** | 8.42 | *b) AR vs others* | 14.14 | ***0.001*** | 7.83 |
| *b) A vs N* | 1 | 2.84 | ***0.001*** | 2.04 | *b) A vs N* | 12.79 | ***0.001*** | 7.08 |
| *c) N vs others* | 1 | 5.18 | ***0.001*** | 3.71 | *c) N vs others* | 20.36 | ***0.001*** | 11.27 |
| *c) AR vs A* | 1 | 9.4 | ***0.001*** | 6.74 | *c) AR vs A* | 6.57 | ***0.001*** | 3.64 |
| Group *A* families (Poaceae vs. Solanaceae) | 1 | 5.59 | ***0.001*** | 4.01 | Group *A* families (Poaceae vs. Solanaceae) | 9.23 | ***0.001*** | 5.11 |
| Group *N* families (Amaranthaceae vs. Brassicaceae) | 1 | 3.34 | ***0.001*** | 2.4 | Group *N* families (Amaranthaceae vs. Brassicaceae) | 17.82 | ***0.001*** | 9.87 |
| Plant sp. within group *A* | 4 | 2.96 | ***0.001*** | 8.49 | Plant sp. within group *A* | 4.49 | ***0.001*** | 9.94 |
| Plant sp. within group *AR* | 4 | 3.42 | ***0.001*** | 9.82 | Plant sp. within group *AR* | 2.49 | ***0.001*** | 5.52 |
| Plant sp. within group *N* | 3 | 2.43 | ***0.001*** | 5.22 | Plant sp. within group *N* | 0.81 | 0.714 | 1.35 |
| Residuals | 72 | | | 51.64 | Residuals | | | 39.86 |

**Table S5:** ANOVA table testing for differences in primary symbiont OTU relative abundance by symbiotype group. Three different contrasts testing all possible combinations of factor levels of symbiotic groups against each other were performed (a, b, c). ANOVA models were tested against the residual error.

| **Bacterial Primary Symbionts** | **df** | **F** | ***p*** | **%-SS** |
|---|---|---|---|---|
| seqDepth | *1* | 7.62 | ***0.007*** | 2.57 |
| Pot | *1* | 0.01 | 0.908 | 0 |
| isLupin | *1* | 8.5 | ***0.005*** | 2.87 |
| Symbiotic Group | *2* | 45.37 | ***0.001*** | 30.58 |
| a) *A* vs others | *1* | 43.28 | ***0.001*** | 14.59 |
| a) *AR* vs *N* | *1* | 47.46 | ***0.001*** | 15.99 |
| b) *AR* vs others | *1* | 88.48 | ***0.001*** | 29.82 |
| b) *A* vs *N* | *1* | 2.26 | 0.137 | 0.76 |
| c) *N* vs others | *1* | 18.45 | ***0.001*** | 6.22 |
| c) *AR* vs *A* | *1* | 72.3 | ***0.001*** | 24.37 |
| Plant species | *13* | 9.06 | ***0.001*** | 39.71 |
| Residuals | *72* | | | 24.27 |
| | | | | |
| **Fungal Primary Symbionts** | **df** | **F** | ***p*** | **%-SS** |
| seqDepth | *1* | 48.91 | ***0.001*** | 13.79 |
| Pot | *1* | 0.86 | 0.357 | 0.24 |
| isLupin | *1* | 8.3 | ***0.005*** | 2.34 |
| Symbiotic Group | *2* | 49.19 | ***0.001*** | 27.73 |
| a) *A* vs others | *1* | 1.99 | 0.163 | 0.56 |
| a) *AR* vs *N* | *1* | 96.39 | ***0.001*** | 27.17 |
| b) *AR* vs others | *1* | 52.32 | ***0.001*** | 14.75 |
| b) *A* vs *N* | *1* | 46.06 | ***0.001*** | 12.98 |
| c) *N* vs others | *1* | 95.66 | ***0.001*** | 26.97 |
| c) *AR* vs *A* | *1* | 2.72 | 0.104 | 0.77 |
| Plant species | *13* | 9.71 | ***0.001*** | 35.6 |
| Residuals | *72* | | | 20.3 |

**Table S6**: ANOVA table testing for the effects of symbiotic groups, plant family, and plant species on bacterial and fungal species richness, effective species richness, and Pielou's evenness for the dataset with primary symbionts removed. For the term "Symbiotic Group", three different contrasts testing all possible combinations of factor levels of symbiotic groups against each other were performed (a, b, c).

| | | **Observed Richness** | | | **Effective Richness** | | | **Evenness** | | |
|---|---|---|---|---|---|---|---|---|---|---|
| **Bacterial OTUs** | df | **F** | **P** | **%-SS** | **F** | **P** | **%-SS** | **F** | **P** | **%-SS** |
| seqDepth | *1* | 30.16 | ***<0.001*** | 22.81 | 1.57 | 0.214 | 1.25 | 11.38 | ***0.001*** | 8.59 |
| Pot | *1* | 0.39 | 0.537 | 0.29 | 13.20 | ***0.001*** | 10.53 | 12.86 | ***0.001*** | 9.7 |
| isLupin | *1* | 1.49 | 0.226 | 1.13 | 0.00 | 0.979 | 0 | 0.41 | 0.522 | 0.31 |
| Symbiotic Group | *2* | 4.33 | ***0.017*** | 6.56 | 0.01 | 0.994 | 0.01 | 1.18 | 0.313 | 1.78 |
| *a) A vs others* | *1* | 7.93 | ***0.006*** | 6 | 0.01 | 0.931 | 0.01 | 1.52 | 0.222 | 1.14 |
| *a) AR vs N* | *1* | 0.73 | 0.394 | 0.56 | 0 | 0.950 | 0 | 0.85 | 0.360 | 0.64 |
| *b) AR vs others* | *1* | 5.31 | ***0.024*** | 4.02 | 0.01 | 0.920 | 0.01 | 2.12 | 0.150 | 1.6 |
| *b) A vs N* | *1* | 3.35 | 0.071 | 2.54 | 0 | 0.972 | 0 | 0.24 | 0.625 | 0.18 |
| *c) N vs others* | *1* | 0.02 | 0.895 | 0.01 | 0 | 0.976 | 0 | 0.21 | 0.649 | 0.16 |
| *c) AR vs A* | *1* | 8.65 | ***0.004*** | 6.54 | 0.01 | 0.918 | 0.01 | 2.15 | 0.147 | 1.63 |
| Group *A* families (Poaceae vs. Solanaceae) | *1* | 1.12 | 0.293 | 0.85 | 3.24 | 0.076 | 2.58 | 2.14 | 0.148 | 1.62 |
| Group *N* families (Amaranthaceae vs. Brassicaceae) | *1* | 0.43 | 0.516 | 0.32 | 0.99 | 0.323 | 0.79 | 0.26 | 0.611 | 0.2 |
| Plant sp. within group *A* | *4* | 0.69 | 0.600 | 2.09 | 2.4 | 0.058 | 7.67 | 2.93 | ***0.027*** | 8.84 |
| Plant sp. within group *AR* | *4* | 1.55 | 0.197 | 4.69 | 0.91 | 0.460 | 2.92 | 0.97 | 0.429 | 2.93 |
| Plant sp. within group *N* | *3* | 2.99 | ***0.036*** | 6.8 | 7.03 | ***<0.001*** | 16.82 | 5.17 | ***0.003*** | 11.7 |
| Residuals | *72* | | | 54.46 | | | 57.43 | | | 54.33 |
| | | **Observed Richness** | | | **Effective Richness** | | | **Evenness** | | |
| **Fungal OTUs** | df | **F** | **P** | **%-SS** | **F** | **P** | **%-SS** | **F** | **P** | **%-SS** |
| seqDepth | *1* | 2.88 | 0.094 | 2.19 | 64.51 | ***<0.001*** | 33.77 | 106.53 | ***<0.001*** | 37.77 |
| Pot | *1* | 0 | 0.967 | 0 | 0.95 | 0.333 | 0.5 | 9.48 | ***0.003*** | 3.36 |
| isLupin | *1* | 2.92 | 0.092 | 2.22 | 0.21 | 0.644 | 0.11 | 0.86 | 0.356 | 0.31 |
| Symbiotic Group | *2* | 5.3 | ***0.007*** | 8.06 | 8.59 | ***0.001*** | 9 | 24.91 | ***0.001*** | 17.67 |
| *a) A vs others* | *1* | 7.06 | ***0.010*** | 5.37 | 4.6 | ***0.035*** | 2.41 | 6.22 | ***0.015*** | 2.21 |
| *a) AR vs N* | *1* | 3.54 | 0.064 | 2.69 | 12.59 | ***0.001*** | 6.59 | 43.61 | ***0.001*** | 15.46 |
| *b) AR vs others* | *1* | 0 | 0.987 | 0 | 2.81 | 0.098 | 1.47 | 15.83 | ***0.002*** | 5.61 |
| *b) A vs N* | *1* | 10.6 | ***0.002*** | 8.06 | 14.37 | ***0.001*** | 7.52 | 34 | ***0.003*** | 12.05 |
| *c) N vs others* | *1* | 6.77 | ***0.011*** | 5.15 | 16.26 | ***0.001*** | 8.51 | 49.7 | ***0.004*** | 17.62 |
| *c) AR vs A* | *1* | 3.83 | 0.054 | 2.92 | 0.92 | 0.340 | 0.48 | 0.13 | 0.718 | 0.05 |
| Group *A* families (Poaceae vs. Solanaceae) | *1* | 0.06 | 0.803 | 0.05 | 5.36 | ***0.023*** | 2.81 | 0.99 | 0.324 | 0.35 |
| Group *N* families (Amaranthaceae vs. Brassicaceae) | *1* | 22.38 | ***<0.001*** | 17.02 | 5.35 | ***0.024*** | 2.8 | 28.18 | ***<0.001*** | 9.99 |
| Plant sp. within group *A* | *4* | 0.89 | 0.474 | 2.71 | 5.01 | ***0.001*** | 10.49 | 1.81 | 0.136 | 2.57 |
| Plant sp. within group *AR* | *4* | 1.31 | 0.273 | 4 | 0.44 | 0.782 | 0.91 | 0.19 | 0.942 | 0.27 |
| Plant sp. within group *N* | *3* | 3.94 | ***0.012*** | 8.99 | 1.22 | 0.309 | 1.92 | 2.04 | 0.115 | 2.17 |
| Residuals | *72* | | | 54.76 | | | 37.69 | | | 25.53 |

**Table S7:** Significantly different bacterial and fungal OTUs between the different groupings of symbiotic status of plants for the dataset with primary symbionts removed. Values reflect the number of OTUs that were identified as being significantly different in the contrasts shown in the first column. The columns 'less abundant' and 'more abundant' refer to the number of OTUs that were significantly less or more abundant in the first group of the indicated contrast.

| **Bacterial OTUs – 4,275 tested** | # less abundant OTUs | # more abundant OTUs | # total significant OTUs | % significant OTUs |
|---|---|---|---|---|
| Rhizobia (*N & A* vs. *AR & R*) | 470 | 180 | 650 | 15.2 |
| Rhizobia, within +AMF plants (*A* vs. *AR*) | 309 | 113 | 422 | 9.87 |
| AMF (*N & R vs. A & AR*) | 211 | 146 | 357 | 8.35 |
| AMF, within -rhizo. Plants (*N vs A*) | 121 | 125 | 246 | 5.75 |
| Between +AMF +rhizo. and -AMF -rhizo plant species (*AR* vs. *N*) | 347 | 157 | 504 | 11.97 |
| ***Unique siginificant OTUs across all tests*** | | | ***953*** | ***22.29*** |
| **Fungal OTUs – 361 tested** | # less abundant OTUs | # more abundant OTUs | # total significant OTUs | % significant OTUs |
| Rhizobia (*N & A* vs. *AR & R*) | 40 | 14 | 54 | 14.96 |
| Rhizobia, within +AMF plants (*A* vs. *AR*) | 35 | 4 | 39 | 10.8 |
| AMF (*N & R vs. A & AR*) | 14 | 27 | 41 | 11.36 |
| AMF, within -rhizo. Plants (*N vs A*) | 10 | 25 | 35 | 9.7 |
| Between +AMF +rhizo. and -AMF -rhizo plant species (*AR* vs. *N*) | 21 | 12 | 33 | 9.14 |
| ***Unique siginificant OTUs across all tests*** | | | ***83*** | ***22.99*** |

**Table S8**: Symbiotic group enriched bacterial and fungal OTUs for the three different symbiosis groups with the primary symbiont OTUs removed. The values in the table indicate the number of OTUs belonging to the indicated taxonomic group that were determined to be significantly enriched in the different symbiotic groups.

| **Bacterial OTUs** | | | | | | | |
|---|---|---|---|---|---|---|---|
| **Phylum** | **Class** | **Order** | **Family** | **Genus** | **N** | **A** | **AR** |
| *Proteobacteria* | *Alphaproteobacteria* | *Caulobacterales* | na | na | 1 | 2 | 1 |
| *Proteobacteria* | *Betaproteobacteria* | *Methylophilales* | na | na | 0 | 0 | 2 |
| *Proteobacteria* | *Gammaproteobacteria* | *Pseudomonadales* | na | na | 0 | 1 | 1 |
| *Proteobacteria* | *Betaproteobacteria* | *Burkholderiales* | na | na | 21 | 6 | 9 |
| *Candidatus_Saccharibacteria* | *Saccharibacteria* | *Saccharibacteria_* | na | na | 1 | 0 | 1 |
| *Proteobacteria* | *Alphaproteobacteria* | *Rhizobiales* | na | na | 5 | 9 | 12 |
| *Acidobacteria* | *Acidobacteria_Gp2* | *undef_undef_Gp2* | na | na | 0 | 1 | 0 |
| *Bacteroidetes* | *Flavobacteriia* | *Flavobacteriales* | na | na | 8 | 0 | 1 |
| *Proteobacteria* | *Deltaproteobacteria* | ukn | na | na | 1 | 0 | 0 |
| *Proteobacteria* | *Alphaproteobacteria* | *Sphingomonadales* | na | na | 6 | 5 | 8 |
| *Proteobacteria* | *Gammaproteobacteria* | *Xanthomonadales* | na | na | 7 | 0 | 1 |
| *Actinobacteria* | *Actinobacteria* | *Actinomycetales* | na | na | 14 | 8 | 2 |
| *Proteobacteria* | *Betaproteobacteria* | *Rhodocyclales* | na | na | 1 | 0 | 1 |
| *Proteobacteria* | *Deltaproteobacteria* | *Myxococcales* | na | na | 4 | 3 | 0 |
| *Proteobacteria* | *Alphaproteobacteria* | *Rhodospirillales* | na | na | 0 | 1 | 1 |
| *Bacteroidetes* | *Sphingobacteriia* | *Sphingobacteriales* | na | na | 1 | 0 | 5 |
| *Proteobacteria* | *Gammaproteobacteria* | ukn | na | na | 1 | 1 | 1 |
| *Proteobacteria* | *Betaproteobacteria* | ukn | na | na | 1 | 0 | 1 |
| *Actinobacteria* | *Actinobacteria* | *Solirubrobacterales* | na | na | 0 | 0 | 1 |
| *Proteobacteria* | ukn | ukn | na | na | 1 | 6 | 2 |
| *Firmicutes* | *Bacilli* | *Bacillales* | na | na | 0 | 4 | 2 |
| *Bacteroidetes* | ukn | ukn | na | na | 1 | 0 | 0 |
| *Bacteroidetes* | *Cytophagia* | *Cytophagales* | na | na | 2 | 2 | 1 |
| *Proteobacteria* | *Alphaproteobacteria* | ukn | na | na | 2 | 7 | 3 |

| | | | | | | | |
|---|---|---|---|---|---|---|---|
| ukn | ukn | ukn | na | na | 1 | 16 | 1 |
| *Proteobacteria* | *Betaproteobacteria* | *Hydrogenophilales* | na | na | 1 | 0 | 0 |
| *Armatimonadetes* | ukn | ukn | na | na | 0 | 1 | 0 |
| *Planctomycetes* | *Planctomycetia* | *Planctomycetales* | na | na | 0 | 1 | 0 |
| *Parcubacteria* | *Parcubacteria* | *Parcubacteria* | na | na | 0 | 1 | 0 |
| **Total** | | | | | **80** | **75** | **57** |
| **Fungal OTUs** | | | | | | | |
| *Ascomycota* | *Dothideomycetes* | *Capnodiales* | *Cladosporiaceae* | *Cladosporium* | 0 | 1 | 0 |
| *Ascomycota* | *Dothideomycetes* | *Pleosporales* | *Phaeosphaeriaceae* | *Paraphoma* | 0 | 0 | 1 |
| *Ascomycota* | *Dothideomycetes* | *Pleosporales* | *Pleosporaceae* | ukn | 0 | 1 | 0 |
| *Ascomycota* | *Dothideomycetes* | *Pleosporales* | ukn | ukn | 0 | 1 | 0 |
| *Ascomycota* | *Sordariomycetes* | *Glomerellales* | *Plectosphaerellaceae* | *Plectosphaerella* | 1 | 0 | 0 |
| *Ascomycota* | *Sordariomycetes* | *Glomerellales* | *Plectosphaerellaceae* | *Verticillium* | 1 | 0 | 0 |
| *Ascomycota* | *Sordariomycetes* | *Hypocreales* | *Bionectriaceae* | *Gliomastix* | 1 | 0 | 0 |
| *Ascomycota* | *Sordariomycetes* | *Magnaporthales* | *Magnaporthaceae* | *Slopeiomyces* | 0 | 1 | 0 |
| *Ascomycota* | *Sordariomycetes* | *Sordariales* | *Chaetomiaceae* | ukn | 1 | 0 | 0 |
| *Ascomycota* | *Sordariomycetes* | *Sordariales* | *Sordariaceae* | ukn | 1 | 0 | 0 |
| *Ascomycota* | ukn | ukn | ukn | ukn | 0 | 2 | 1 |
| *Basidiomycota* | *Tremellomycetes* | *Trichosporonales* | *Trichosporonaceae* | *Trichosporon* | 0 | 1 | 0 |
| *Olpidiomycota* | *Olpidiomycetes* | *Olpidiales* | *Olpidiaceae* | *Olpidium* | 1 | 0 | 0 |
| ukn | ukn | ukn | ukn | ukn | 3 | 7 | 2 |
| **Total** | | | | | **9** | **14** | **4** |
